# A New Approach for Detecting Sleep Apnea Using a Contactless Bed Sensor: Comparison Study

**DOI:** 10.2196/18297

**Published:** 2020-09-18

**Authors:** Ibrahim Sadek, Terry Tan Soon Heng, Edwin Seet, Bessam Abdulrazak

**Affiliations:** 1 AMI-Lab, Computer Science Department, Faculty of Science University of Sherbrooke Sherbrooke, QC Canada; 2 Research Centre on Aging Sherbrooke, QC Canada; 3 Biomedical Engineering Dept, Faculty of Engineering Helwan University Helwan, Cairo Egypt; 4 Department of Otolaryngology, Woodlands Health Campus and Khoo Teck Puat Hospital Singapore Singapore; 5 Department of Anaesthesia, Khoo Teck Puat Hospital Singapore Singapore

**Keywords:** ballistocardiography, sleep apnea, vital signs, eHealth, mobile health, home care

## Abstract

**Background:**

At present, there is an increased demand for accurate and personalized patient monitoring because of the various challenges facing health care systems. For instance, rising costs and lack of physicians are two serious problems affecting the patient’s care. Nonintrusive monitoring of vital signs is a potential solution to close current gaps in patient monitoring. As an example, bed-embedded ballistocardiogram (BCG) sensors can help physicians identify cardiac arrhythmia and obstructive sleep apnea (OSA) nonintrusively without interfering with the patient’s everyday activities. Detecting OSA using BCG sensors is gaining popularity among researchers because of its simple installation and accessibility, that is, their nonwearable nature. In the field of nonintrusive vital sign monitoring, a microbend fiber optic sensor (MFOS), among other sensors, has proven to be suitable. Nevertheless, few studies have examined apnea detection.

**Objective:**

This study aims to assess the capabilities of an MFOS for nonintrusive vital signs and sleep apnea detection during an in-lab sleep study. Data were collected from patients with sleep apnea in the sleep laboratory at Khoo Teck Puat Hospital.

**Methods:**

In total, 10 participants underwent full polysomnography (PSG), and the MFOS was placed under the patient’s mattress for BCG data collection. The apneic event detection algorithm was evaluated against the manually scored events obtained from the PSG study on a minute-by-minute basis. Furthermore, normalized mean absolute error (NMAE), normalized root mean square error (NRMSE), and mean absolute percentage error (MAPE) were employed to evaluate the sensor capabilities for vital sign detection, comprising heart rate (HR) and respiratory rate (RR). Vital signs were evaluated based on a 30-second time window, with an overlap of 15 seconds. In this study, electrocardiogram and thoracic effort signals were used as references to estimate the performance of the proposed vital sign detection algorithms.

**Results:**

For the 10 patients recruited for the study, the proposed system achieved reasonable results compared with PSG for sleep apnea detection, such as an accuracy of 49.96% (SD 6.39), a sensitivity of 57.07% (SD 12.63), and a specificity of 45.26% (SD 9.51). In addition, the system achieved close results for HR and RR estimation, such as an NMAE of 5.42% (SD 0.57), an NRMSE of 6.54% (SD 0.56), and an MAPE of 5.41% (SD 0.58) for HR, whereas an NMAE of 11.42% (SD 2.62), an NRMSE of 13.85% (SD 2.78), and an MAPE of 11.60% (SD 2.84) for RR.

**Conclusions:**

Overall, the recommended system produced reasonably good results for apneic event detection, considering the fact that we are using a single-channel BCG sensor. Conversely, satisfactory results were obtained for vital sign detection when compared with the PSG outcomes. These results provide preliminary support for the potential use of the MFOS for sleep apnea detection.

## Introduction

### Monitoring of Contactless Patients

At present, there are many hurdles confronting health care providers and decision makers, such as the sizable aging patient population, the rising prevalence of chronic diseases, the ever-growing health care spending, and the shortage of clinicians [1,2]. To emphasize, the Association of American Medical Colleges anticipates that the United States could face a shortage of 122,000 physicians by 2032 as the need for physicians outpaces supply [3]. Thus, physicians may not achieve close and continuous monitoring of chronically ill patients on time, thereby increasing their rate of mortality [4]. Apart from ongoing challenges, the existing modalities used to monitor patients at the hospital are too intrusive. They require attaching sensors to the skin or strapping devices to the body. As a result, they will have limited benefits outside hospital rooms. In other words, patients are not monitored before and after being admitted to the hospital. 

By comparison, remote and continuous monitoring of patients through contactless sensors can effectively assist physicians in keeping track of their patients’ health status while they are at home. More importantly, monitoring and managing patient populations with chronic diseases in a contactless way is essential to avoid additional distress. Contactless monitoring can be achieved largely because of the miniaturization of microprocessors, which allows researchers to integrate sensors into familiar objects, for example, home appliances and mobile devices [5]. Infrared motion sensors, for instance, can capture patients’ indoor activities such as being still/moving and moving across rooms. Similarly, contact sensors can capture room, cupboards, and fridge door opening and/or closing. Bed-embedded sensors, also known as ballistocardiogram (BCG) sensors, can deliver noteworthy information about the patient’s vital signs, that is, heart rate (HR), breathing, body movements, and quality of sleep [6]. In all, researchers, through contactless sensors, are ultimately trying to predict changes in a patient’s health status that can prevent or delay the progression of diseases [7,8]. The hypothesis is that the health status of patients admitted to hospitals is not suddenly deteriorating. Monitoring vital sign trends over time can provide early diagnosis and allow physicians or caregivers to make timely decisions [9]. In this study, we introduce a new approach using a contactless system that is based on the ballistocardiographic principle for detecting abnormal breathing events (ie, apneas and hypopneas) in an effort to address one of today’s health care issues.

### Sleep Apnea Facts and Diagnoses

The most common form of sleep-disordered breathing is obstructive sleep apnea (OSA). It occurs when a complete or partial closure of the upper airway triggers apnea and hypopnea during sleep [10]. An apnea is a cessation of breathing for at least 10 seconds. Hypopnea is a reduction in airflow for at least 10 seconds by at least 30% accompanied by a drop in oxygen saturation and/or arousal from sleep [11]. Among the general public, OSA affects both men (34%) and women (17%). Nonetheless, it is believed that the prevalence of this syndrome might be underrated. To illustrate, in the United States, estimates showed that 82% of men and 93% of women are underdiagnosed [12].

OSA severity is determined in reference to the apnea-hypopnea index (AHI), that is, the average number of apnea and hypopnea episodes observed per hour of sleep. The severity of OSA is classified as follows: normal (no OSA; AHI <5 events per hour), mild sleep apnea (AHI ≥5 and <15 events per hour), moderate sleep apnea (AHI ≥15 and <30 events per hour), and severe sleep apnea (AHI ≥30 events per hour) [13]. In this regard, patients with moderate or severe apnea are at a higher risk of complications, such as stroke, hypertension, congestive heart failure, and depression. Overall, the late diagnosis of OSA has been shown to double the mortality risk for patients diagnosed with heart failure [14]. The gold standard for evaluating the severity of OSA is polysomnography (PSG). PSG is an overnight controlled sleep study in a specialist sleep laboratory that follows established scoring guidelines for OSA-associated respiratory events.

Through PSG testing, physicians can record different bodily functions. These functions involve HR and rhythms, brain waves, eye movements, leg movements, nasal-oral airflow, thoracoabdominal effort, oxygen saturation, snoring, and body position. The PSG test provides physicians with information about body functions, and therefore, they can diagnose various sleep disorders. However, there are some cons related to the test, for example, high cost, labor intensive, complex, and insufficient privacy. Furthermore, it is not possible to emulate the usual sleep environment in a sleep laboratory. As a consequence, home sleep apnea tests (HSATs) have become alternative possibilities for patients who want to circumvent the in-laboratory PSG. These kinds of tests do not record the full range of signals similar to the PSG. However, they can record up to 7 parameters, including airflow (thermal and nasal pressure), effort (inductive plethysmography), and oximetry [12]. Although such testing is not as reliable as PSG, its portability, affordability, and long-term data collection make it a preferred choice for patients. Recently, off-the-shelf BCG sensors have been investigated by researchers to detect apneic events under the HSAT category. Although the results were encouraging, much work is still needed to reach agreeable results compared with PSG [15]. In this regard, we will discuss later, in brief, the concept of BCG and how it has been employed in the scientific literature to identify apneic events.

### Ballistocardiography and Contactless Apnea Detection–Related Work

Ballistocardiography reflects the movement of the center of mass of the body because of cardiovascular activity. The concept of BCG is not new, and there has been a resurgence because of recent improvements in digital electronics reaching the era of microprocessors. Formerly, BCG systems (ie, tables employed by Starr et al [16]) were bulky, heavy, and complicated, demanding professional mechanical maintenance. Consequently, these systems were principally intended for a single-snapshot recording instead of a long-term data recording [17]. At present, BCG signals are seamlessly being recorded using different sensing modalities, particularly bed-embedded sensors (eg, microbend fiber optic sensors [MFOSs], piezoelectric polyvinylidene sensors, electromechanical film sensors, pneumatic sensors, strain gauges, and hydraulic sensors) [6,18], accelerometers [17,19], and Doppler radar-based sensors [20,21], smart beds [22]. Bed-based sensors, along with accelerometers, can be integrated with everyday objects such as pillows, mattresses, chairs or even installed on the seat of a standard toilet [23]. Moreover, attempts have been made to measure BCG signals via video recording by tracking the motion of facial features [24,25]. Video-based approaches can be practical for surveillance; however, they can impose privacy issues for in-home patient monitoring. Typically, BCG sensors are positioned under the patient’s mattress covering the upper half of the body, which allows capturing heart movements, breathing movements, and overall body movements.

Several publications in the literature highlight the extensive use of BCG sensors for both HR and respiratory rate (RR) detection [6,18], which, in turn, attracted researchers to investigate the benefits of BCG signals for more complicated health issues, namely, cardiovascular functions [26] as well as sleep quality [15]. Regarding sleep quality health issues, efforts have been made in the literature to automate the detection of both sleep staging [27,28] and sleep apnea. So far, there have been a few studies that targeted sleep apnea detection through BCG sensors. In this section, we will focus on sleep apnea detection–related work. The study by Sadek et al [6] has more research on HR detection and/or RR detection.

Tenhunen et al [29] investigated the potential of an electromechanical film-based sensor for diagnosing OSA. Although a high sensitivity was reached for detecting apneic events, breathing patterns were analyzed manually by 2 independent scorers, and no contributions were made to computerize the detection process. Hwang et al [30] proposed the use of a polyvinylidene film-based sensor for detecting apneic events. A rule-based framework was implemented to detect apneic events by considering the SD of the sensor signals. Beattie et al [31] tested the effectiveness of using load cells placed under the support of a bed for apnea detection. Although satisfactory results were achieved, the detection process was performed manually by an expert. Waltisberg et al [32] deployed a sensor system that consisted of an array of strain gauges to detect apnea and periodic limb movement events. A supervised learning framework comprising a decision fusion method and a measurement fusion method was applied for the classification process. Similarly, Wang et al [33] used a supervised learning framework to detect apneic events via a micromovement sensitive mattress. Multiple time-domain and frequency-domain features were extracted, which were then fed to different classifiers, that is, k-nearest neighbor, random forest, and support vector machine. Hsu et al [34] sought to detect apneic events by integrating 2 fiber optic–based sensors within a pillow as well as a bed mattress. Apnea detection was achieved by applying 2 methods, that is, a drop degree from the baseline and linear regression models through the percentage of the total duration of the respiratory declination. To compute the model parameters, the empirical mode decomposition (EMD) algorithm was used. However, this signal analysis method is time consuming and precisely for computing the corresponding intrinsic mode functions. Moreover, it is sensitive to the mode-mixing problem [35].

The supervised learning-based approaches (used in the studies mentioned earlier) require a considerable amount of accurately annotated data, which can become quite restrictive for noncontrolled settings [36]. Manual annotation can be considered as an issue because the morphology of BCG signals is highly dependent on the measurement device. BCG signals can differ significantly between studies; besides, they differ within and between subjects. Comparatively, Huysmans et al [37] tested a commercial BCG sensor, that is, Emfit QS, for sleep apnea screening. Unlike the work proposed by Tenhunen et al [29], the authors automated the apnea detection process as follows: 2 Emfit sensors were employed, that is, one sensor was placed below the thorax of the patient and the second was placed under the topper. The detection was then completed via an unsupervised clustering method. The assumption was that during abnormal breathing events, there will always be substantial variations in the signal due to chest motions; thus, by locating these artifacts, they could detect apneic events. This approach avoided the limitation of supervised learning. Nevertheless, the sensor locations were compared to achieve an optimal agreement with PSG. In other words, the sensor that was very close to the thorax achieved more favorable results than the other sensor. In our study, we considered an MFOS for detecting vital signs. Fiber optic sensors (FOSs) are usually used as transducers to detect various environmental changes, such as pressure, temperature, and acceleration [38]. Owing to their electromechanical field immunity and high sensitivity to variations in environmental properties such as the strain, FOSs have been adopted to monitor important physiological parameters, for example, pulse rate and RR, which in turn can help detect cardiovascular diseases and respiratory anomalies [39,40]. Among other sensors, MFOSs have proven to be efficient in detecting ballistic forces correlated with heart movements. They are also moderately small, lightweight, and economical. Hence, they become popular in contactless monitoring of vital signs [41]. 

The contribution of our study is two-fold. First, we analyzed the robustness of the MFOSs for the simultaneous detection of HR and breathing rate (BR). Second, we examined the capacities of an MFOS for contactless detection of sleep apneic events versus the gold standard overnight in-laboratory PSG.

## Methods

### Recruitment

This study is approved by the National Healthcare Group Domain-Specific Review Board (NHG DSRB Ref: 2017/01117). A written informed consent form was obtained from all patients before data collection. We completed all the processes, as stated in the guidelines and regulations of the NHG DSRB. We recruited 10 patients diagnosed with OSA and scheduled to undergo a full night PSG in the sleep laboratory at Khoo Teck Puat Hospital. Patient demographics and related medical history are presented in Table 1. The MFOS was placed under the patient’s mattress, and the sensor mat’s data were collected in parallel with the overnight PSG data. We imposed no restriction on the exact location of the sensor mat. However, we notified the nurses to locate the mat in the upper part of the bed so that we could acquire cardiac signals as well as respiratory effort signals. The sensor mat does not add any complications to standard PSG protocols because the mat has its own data storage unit. In addition, it does not add any complexity to the patient being monitored. In addition, data analysis was executed offline to align with the ethics approval for the study. To preserve the anonymity of the patients, acquired data were registered with a unique identifier linked to each patient.

**Table 1:** Demographics and past medical history of recruited patients.

Patients	Gender	BMI (kg/m^2^)	Past medical history	Age (years)	Apnea-hypopnea index	Smoking
1	Female	32.8	Nil	51	36.8	No
2	Male	34	Nil	28	33.7	Yes
3	Male	25.5	Nil	23	32.8	No
4	Male	23	Nil	27	58.3	Unknown
5	Male	25.5	Nil	42	26	Unknown
6	Male	24.8	Nil	33	29	No
7	Male	27.5	Hypertension	49	76.6	Yes
8	Male	33.3	Hypertension and dyslipidemia	43	78.2	No
9	Male	34	Dyslipidemia	61	54.8	No
10	Male	31.2	Nil	29	93.2	No

### Microbend Fiber Optic Sensor

**Figure 1:**
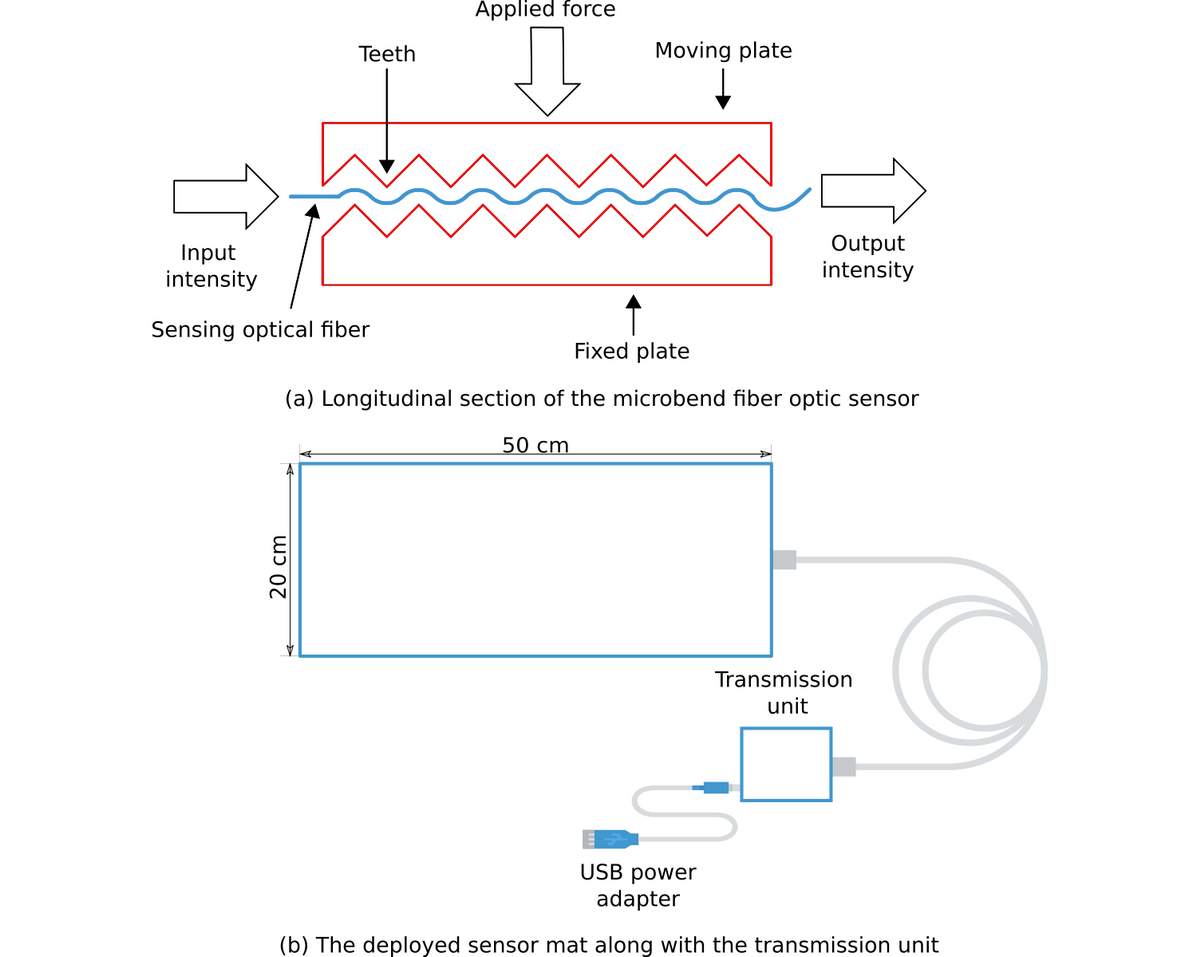
Longitudinal cross-section of the microbend fiber optic sensor along with a schematic diagram of the deployed sensor mat.

The proposed monitoring system incorporates a sensor mat and transmission unit. The sensor mat is assembled to a dimension of 20 cm×50 cm×0.5 cm, which promotes its portability and inclusion into cushions, pillows, chairs, and beds. The transmission unit has a built-in microstorage device card for data storage, digital electronics for signal handling, and a Wi-Fi signal transmission module for sending the data to a cloud-based platform. The deployed sensor employs light-intensity modulation caused by the microbending effect in multimode optical fibers, which can be used as a transduction mechanism for detecting pressure. Further information about the sensor’s working principle can be found in Multimedia Appendix 1. The system was set to collect data at a sampling frequency of 50 Hz. Figure 1 (top) shows a longitudinal cross-section of the deployed MFOS, and Figure 1 (bottom) presents a schematic diagram of the deployed sensor mat.

### Data Analysis

For real-time applications, acquired data are deposited in 5-min chunks on a microstorage device card (4 GB internal storage) consolidated with the transmission unit, and then the chunks are dispatched to a cloud server to extract correlated vital signs. Data chunks are encrypted binary files (BIN), and each file consumes 206 KB. Against this background, data are indecipherable without a proper interpreter. In our application, data chunks were gathered directly from the card. Further information about the structure of the data can be found in Multimedia Appendix 1. The data analysis consisted of 2 stages: (1) vital sign detection and (2) apneic event detection. Ensuing, we describe each stage separately.

#### Vital Sign Detection

The force applied to the sensor mat is the summation of the 3 sources. This force is caused by gross body movements and chest wall movement because of the respiration and cardioballistic effect (BCG) [42]. The BCG signal delivers information about HR and HR variability. Similarly, respiratory signals can report on the RR. Extracting both signals can be completed in different ways, for example, via band-pass filtering, wavelet analysis, or other decomposition methods, namely, EMD. To obtain a successful decomposition, motion artifacts must be suppressed from the raw data. Although they are important indicators of sleep quality, information about vital signs cannot be extracted, as the shape of a typical physiological signal is demolished.

**Figure 2:**
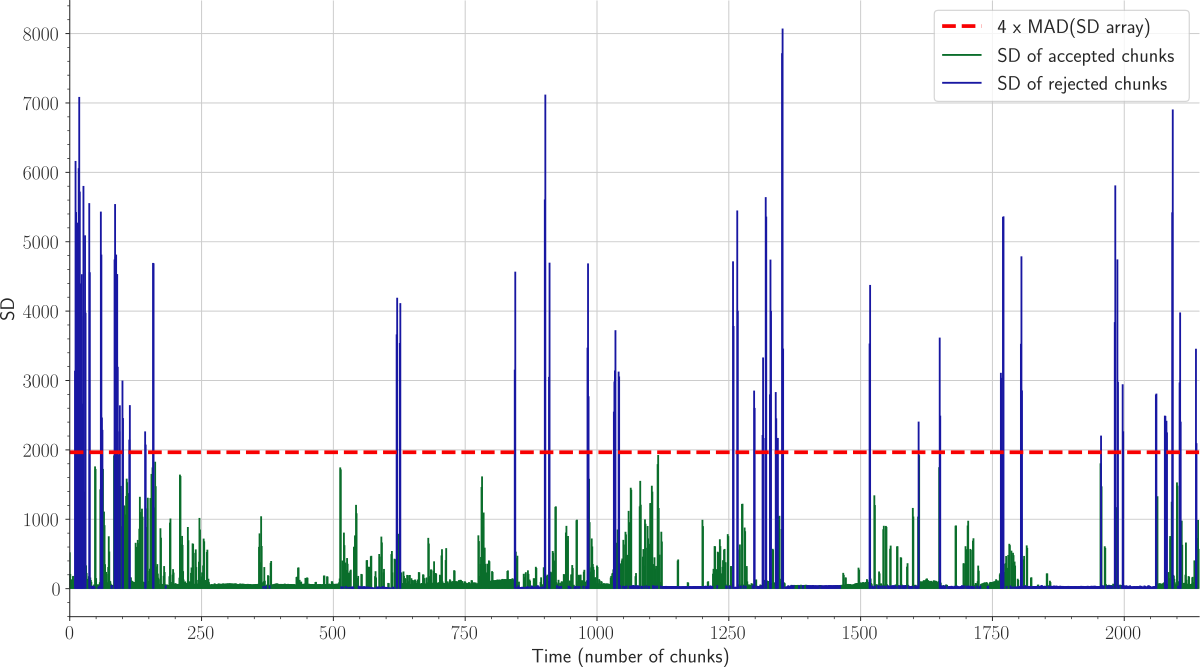
Illustration of isolated motion artifacts. Data chunks were suppressed if they were 4 times greater than the median absolute deviation of the SD array. MAD: median absolute deviation.

Motion artifacts in our approach were suppressed by applying an adaptive threshold method that employed the SD of the raw data [41]. We defined 2 thresholds to remove motion artifacts, that is, *out-of-bed artifact* and *motion artifact*. We divided the raw data stream into equal chunks of 30 seconds, with an overlap of 15 seconds. For each 30-second chunk, we computed the SD and stored all SD values in a single array. Following that, we computed the median absolute deviation (MAD) for the SD array. If the SD of a 30-second chunk was 4 times greater than the MAD, we considered this chunk as a *motion artifact*. In this step, we can control the extent to which the motion artifacts need to be suppressed. When we suppress data chunks with an SD value that is 4 times greater than the MAD, we allow the algorithm to preserve portions of the data with moderately high variation in the signal amplitude (Figure 2). A further increase in this value will allow the algorithm to retain portions of the data with an extremely high variation in the signal amplitude. By selecting this threshold value, we were able to achieve a signal coverage of 79.79%, 81.33%, 78.58%, 84.83%, 86.36%, 87.51%, 81.82%, 51.24%, 75.58%, and 70.59% for all patients, respectively. The coverage is the ratio between the duration of artifact-free signal and raw signal. Ultimately, there should be a balance between the number of recovered signals and the algorithm performance to measure vital signs of interest. If the SD was lower than a predetermined threshold (5 mV), we considered this chunk as an *out-of-bed* activity. This implies that there were no variations in the amplitude of the acquired data. We only calculated HR and RR for data chunks with SD values between these 2 thresholds. HR and RR were detected according to Sadek et al [41], and further information can be found in Multimedia Appendix 1.

**Figure 3:**
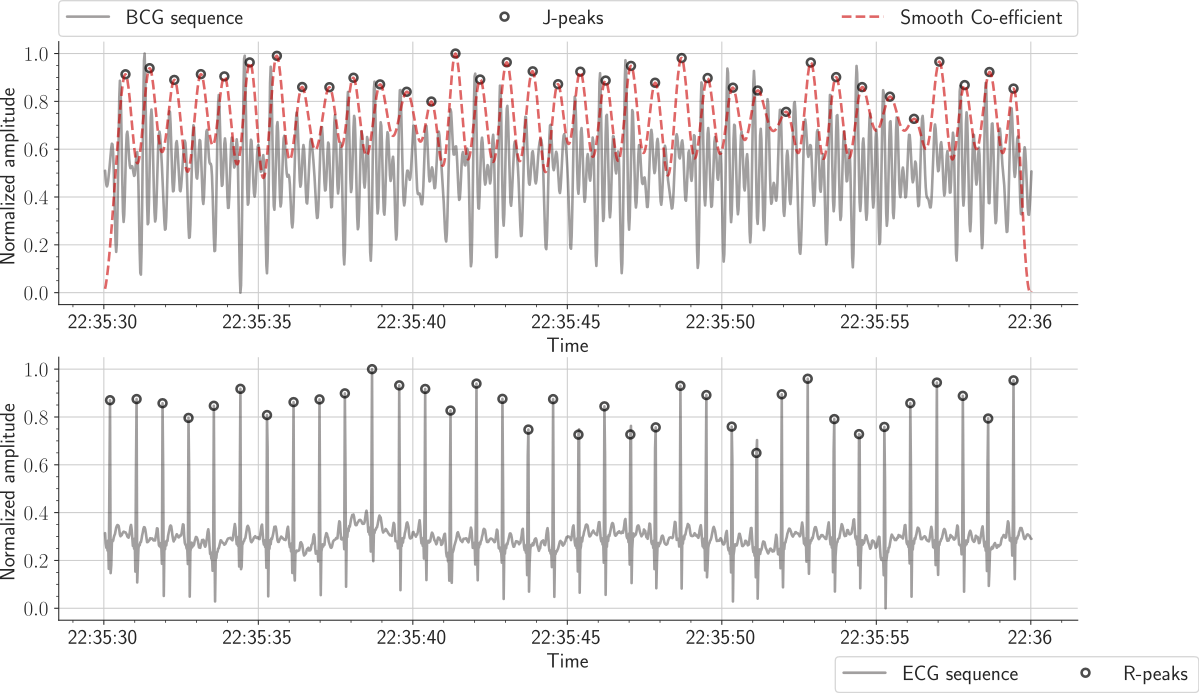
The first row displays a 30-second ballistocardiogram signal and the fourth-level smooth coefficient. The second row displays the equivalent electrocardiogram signal. ECG: electrocardiogram.

**Figure 4:**
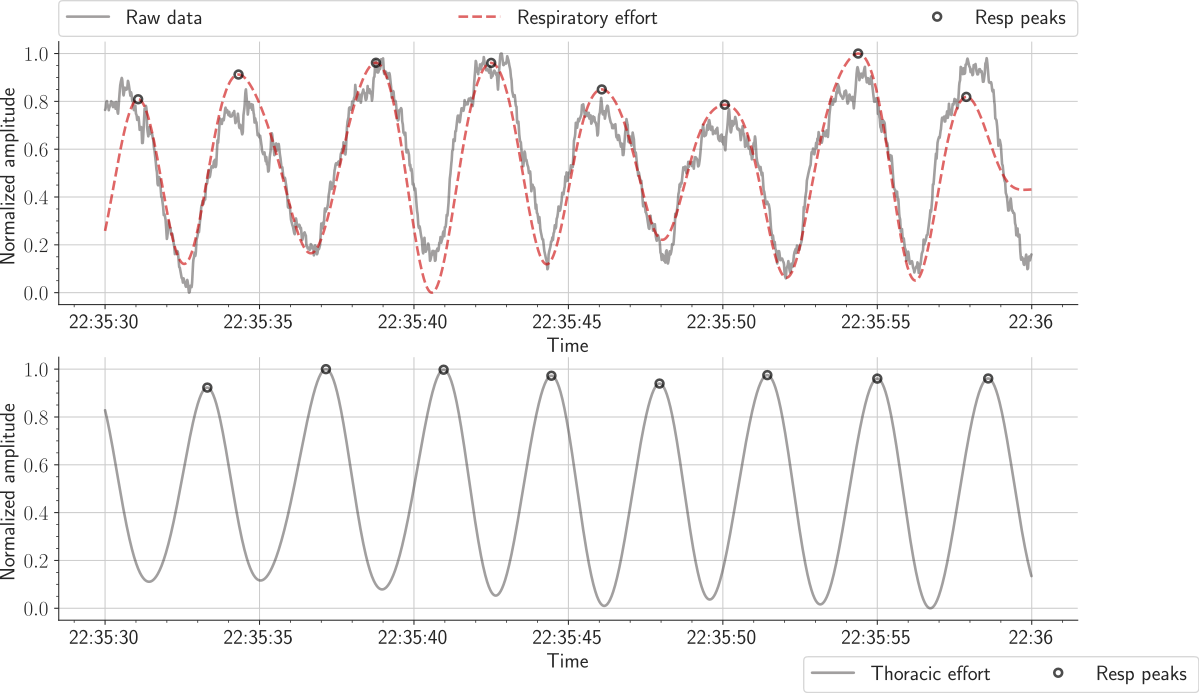
The first row displays a 30-second raw signal and the respiratory effort signal. The second row displays the equivalent thoracic belt signal.

HRs were measured using a sliding time window of 30 seconds, with an overlap of 15 seconds. The electrocardiogram (ECG) signal was used as a reference to detect interbeat intervals (Figure 3). To achieve this objective, we selected the well-known Pan and Tompkins algorithm because of its reasonable results [43]. RRs were calculated using a sliding time window of 30 seconds, with an overlap of 15 seconds (Figure 4). The effort signal obtained from the thoracic belt was used as a reference to detect respiratory cycles. Compared with abdominal effort and airflow (ie, pressure and thermistor) signals, the effort thoracic signal was highly correlated with the signal acquired from the MFOS.

#### Apneic Event Detection

As we quoted earlier, most of the existing methods use supervised learning algorithms to identify apneic events from BCG signals. Although such methods yield favorable results, they impose restrictions because of the morphological variations of the acquired signals. As such, we implemented an adaptive histogram-based thresholding approach for apnea detection. Pauses in breathing must last at least 10 seconds to be counted as apneic events and can last longer depending on the severity of the disease.

**Figure 5:**
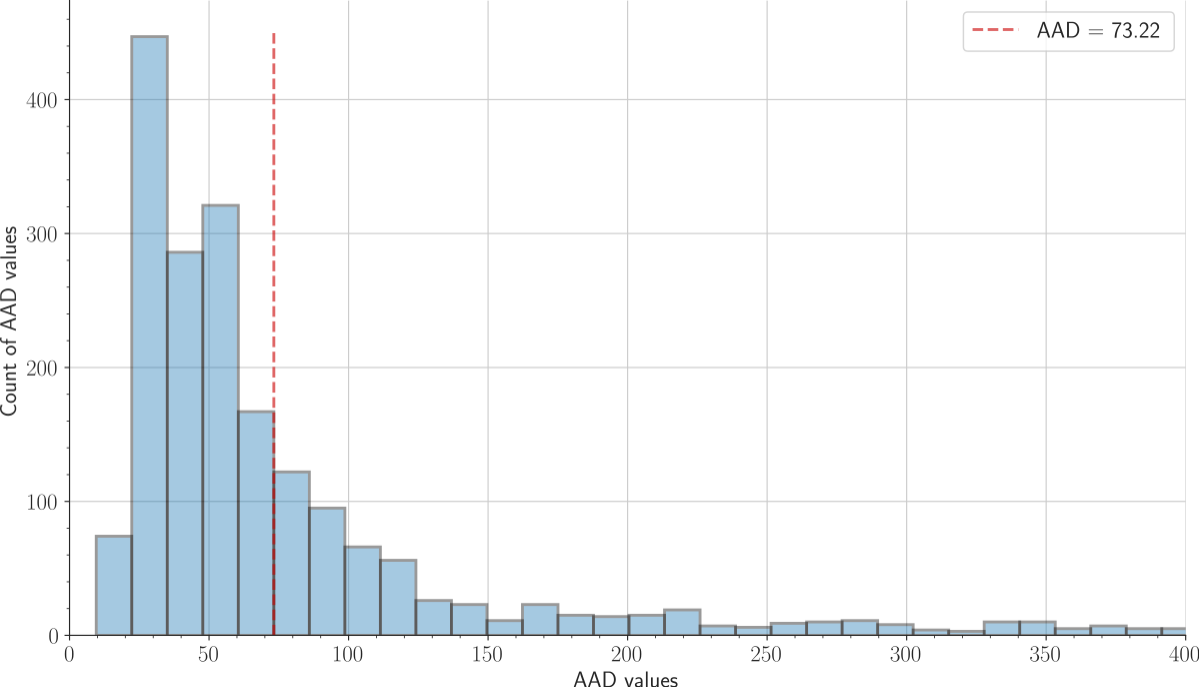
Histogram of the average absolute deviation values for a breathing signal; the gray bars count the average absolute deviation values that fall into each bin. The selected threshold is represented as a red dashed line. The values between 0 and 400 are only displayed to visualize the histogram bins better. AAD: average absolute deviation.

**Figure 6:**
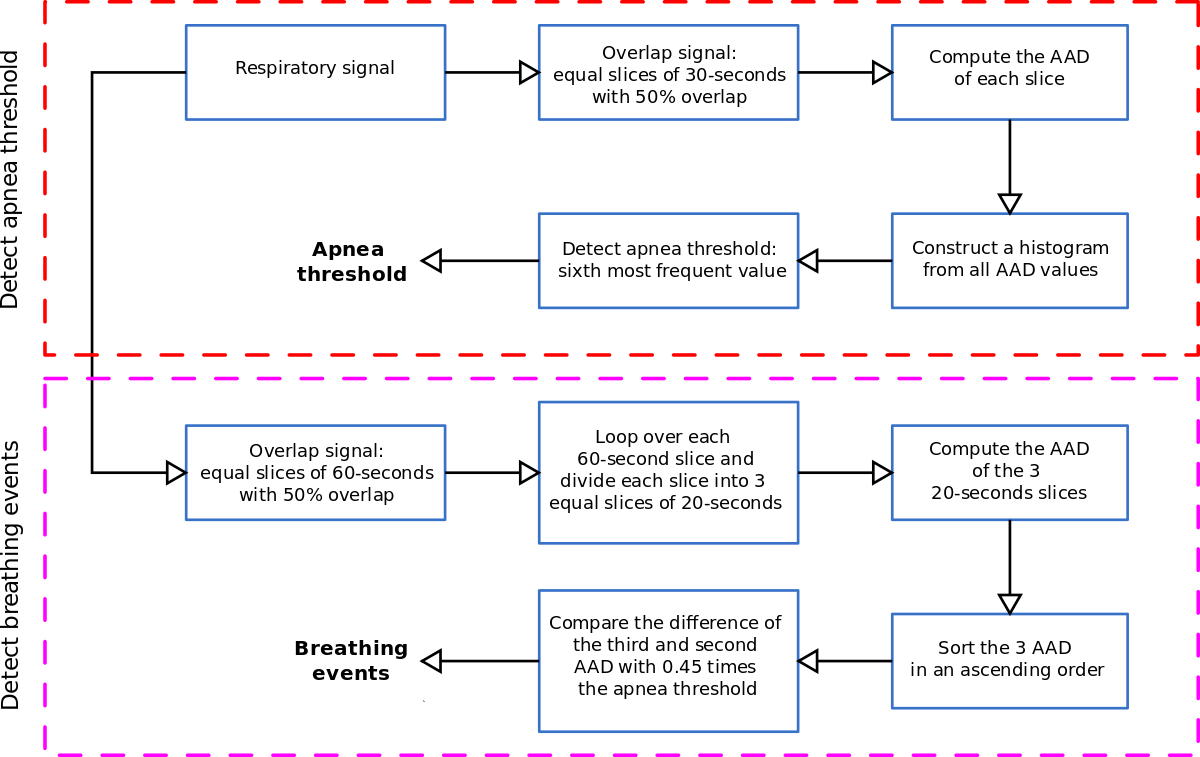
Flowchart of apneic event detection. AAD: average absolute deviation.

These pauses in breathing are accompanied by an increase in the body and breathing movements and snoring. After matching the scored apneic events (ie, PSG manual scoring) with derived breathing signals, we found that most of the apneic events fell during *motion artifact*–labeled slices. Thus, motion artifacts were not removed during apnea detection. In our approach, we aimed to differentiate between apneic and nonapneic events via derived breathing signals. To meet this target for each patient, we constructed a histogram from the average absolute deviation (AAD) of the extracted respiratory signal time windows. The time windows were obtained by slicing the signal into equal slices of 30 seconds, with 50% overlapping; afterward, the histogram (ie, the gray bars in Figure 5) was sorted in descending order. In other words, the first histogram value represented the location of the mode of the AAD values (ie, the AAD value that occurred the most often). The hypothesis was that the most frequent histogram values would correspond to normal breathing events. In this regard, we designated the AAD value equivalent to the 6th histogram value as a threshold to detect apneas, that is, AAD values greater than the selected threshold were assumed to represent apneic events (Figure 5). This histogram value was selected (see the Parameters Selection section) based on the proposed method’s ability to discriminate between normal and apneic events. This value shows consistent results across all patients. After detecting the threshold, we split the breathing signal into equal slices of 60 seconds, with 50% overlapping. Then, every 60-second slice was further split into three 20-second slices. Next, for each 60-second slice, we computed the AAD of the three 20-second slices and stored them in ascending order. If the difference between the third and second elements was greater than 45% (see the Parameters Selection section) of the histogram threshold, we marked the 60-second slice as an apneic event; otherwise, we labeled it as a nonapneic event (Figure 6).

#### Parameter Selection

The proposed method requires the optimization of 2 parameters: a histogram value and a threshold value. A leave-one-out-cross-validation (LOOCV) was implemented to complete this task. The effect of the 2 parameters for apneic event detection was examined in each iteration (ie, 10 iterations in our case) using 9 distinct patient data rather than recording the system’s performance for the held-out patient. A system’s performance via the LOOCV is usually carried out based on the outcome of a held-out point (ie, a patient in our case). Then, the overall performance is computed by taking the average of the evaluation metrics across all iterations. For our study, we capitalized on this approach to choose the optimal values for the 2 specified parameters. First, we aimed to determine the optimal kth histogram value for an arbitrary threshold value. Various threshold values were exploited (range 0.2-0.95, with a step size of 0.05). Similarly, several histograms (2nd histogram to 16th histogram) were tested against each individual threshold. In other words, for a single threshold value, 3 evaluation metrics (ie, sensitivity, specificity, and accuracy) were measured in accordance with 15 histogram values. This process was repeated 10 times using 9 distinct patient data, and in each iteration, the mean of each metric was recorded. The objective of this process was to find an optimal histogram value applicable to any arbitrary threshold.

**Figure 7:**
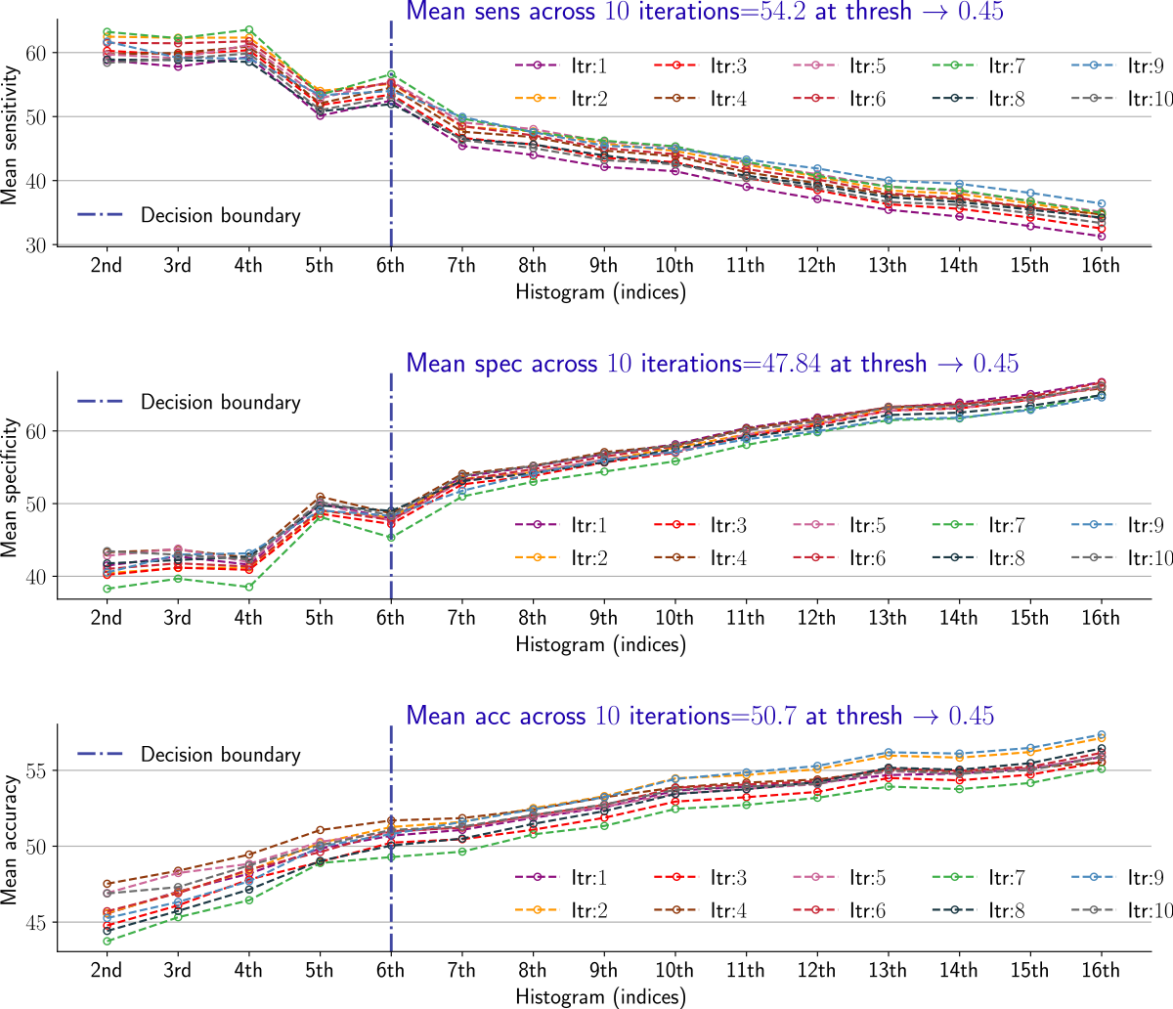
Optimal histogram selection at a 45% threshold value. The 1st row represents the mean sensitivity in each iteration versus different histogram values. The second and third rows represent the specificity and accuracy, respectively.

**Figure 8:**
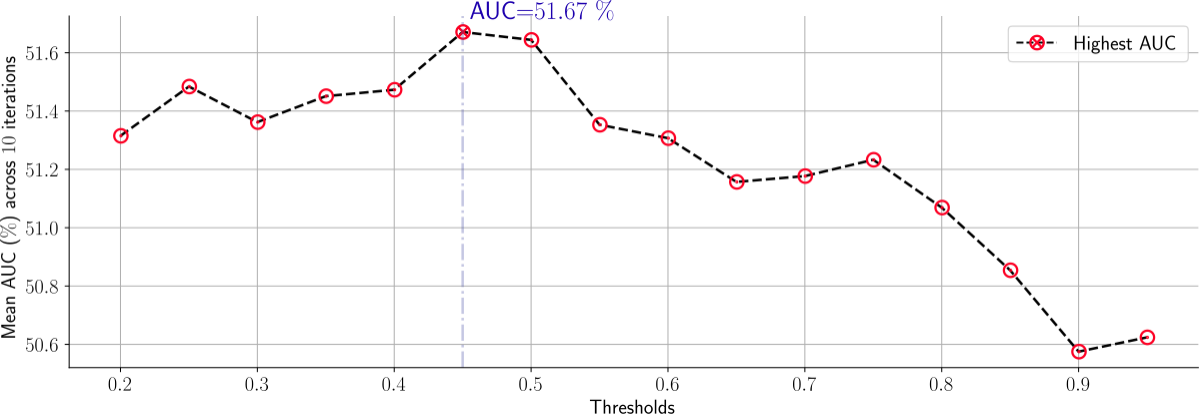
Optimal threshold selection corresponding to the 6th histogram value. The overall mean area under the curve was computed across the 10 iterations and plotted against different threshold values. AUC: area under the curve.

For any threshold value, the sensitivity was inversely proportional to the histogram values. However, the specificity and accuracy were directly proportional to the histogram values. Thus, the 6th histogram value can be considered as a critical point. As shown in Figure 7, there was a rapid change in the sensitivity and specificity between the 5th and 7th histograms. The sensitivity keeps decreasing with small fluctuations beyond the 6th histogram value, whereas the opposite occurred for specificity and accuracy. The same behavior occurred for all arbitrary threshold values. As a result, the 6th histogram value was selected as the optimal value for apneic event detection. Second, we aimed to determine the optimal threshold value compatible with the 6th histogram value. To achieve this task, we computed the overall mean area under the curve (AUC), also called balanced accuracy across the 10 iterations for each individual threshold value. The threshold yielding the highest AUC was selected as the optimal value. As shown in Figure 8, the highest AUC occurred at a 45% threshold value, that is, 51.67%. As a result, the 45% threshold value was selected as the optimal value for apneic event detection.

### Statistical Analysis

For apneic event detection, we compared the apneic events provided by the PSG with those recovered from the advised sensor mat. Different metrics were adopted in performing the appraisal, that is, sensitivity, specificity, and accuracy. On the other hand, HR and RR were assessed in beats per minute and breaths per minute, respectively. To quantify the performance of the proposed sensor mat for HR and BR estimates compared with the reference ECG signal and effort belt signal, the Bland-Altman plot, Pearson correlation coefficient, normalized root mean square error (NRMSE), normalized mean absolute error (NMAE), and mean absolute percentage error (MAPE) were adopted. These metrics are commonly employed to determine the difference between medical instruments [44-48]. Further information about these error metrics can be found in Multimedia Appendix 1. High-quality PNG images of all figures presented in the study can be found in Multimedia Appendix 2.

## Results

### Heart and Respiratory Measurements

On average, the NMAE, NRMSE, and MAPE were 5.42% (SD 0.57), 6.54% (SD 0.56), and 5.41% (SD 0.58) for HR estimation, respectively (Table 2). In addition, the NMAE, NRMSE, and MAPE were 11.42% (SD 2.62), 13.85% (SD 2.78), and 11.60% (SD 2.84), for RR estimation, respectively (Table 3).

**Table 2:** Normalized mean absolute error, normalized root mean square error, and mean absolute percentage error for heart rate estimation.

Patients	Normalized mean absolute error (%)	Normalized root mean square error (%)	Mean absolute percentage error (%)
1	4.20	5.30	4.16
2	5.74	6.91	5.69
3	5.96	7.15	6.02
4	5.28	6.45	5.27
5	5.08	6.19	5.03
6	5.22	6.33	5.20
7	5.66	6.84	5.73
8	6.26	7.19	6.20
9	5.32	6.39	5.29
10	5.45	6.57	5.40
Mean (SD)	5.42 (0.57)	6.54 (0.56)	5.41 (0.58)

**Table 3:** Normalized mean absolute error, normalized root mean square error, and mean absolute percentage error for respiratory rate estimation.

Patients	Normalized mean absolute error (%)	Normalized root mean square error (%)	Mean absolute percentage error (%)
1	8.84	11.34	8.80
2	8.43	10.91	8.54
3	12.76	15.04	12.66
4	14.44	16.93	15.00
5	14.33	16.88	14.86
6	14.69	17.64	15.22
7	8.20	10.28	8.14
8	10.22	12.09	10.28
9	9.74	12.20	9.67
10	12.51	15.21	12.84
Mean (SD)	11.42 (2.62)	13.85 (2.78)	11.60 (2.84)

**Figure 9:**
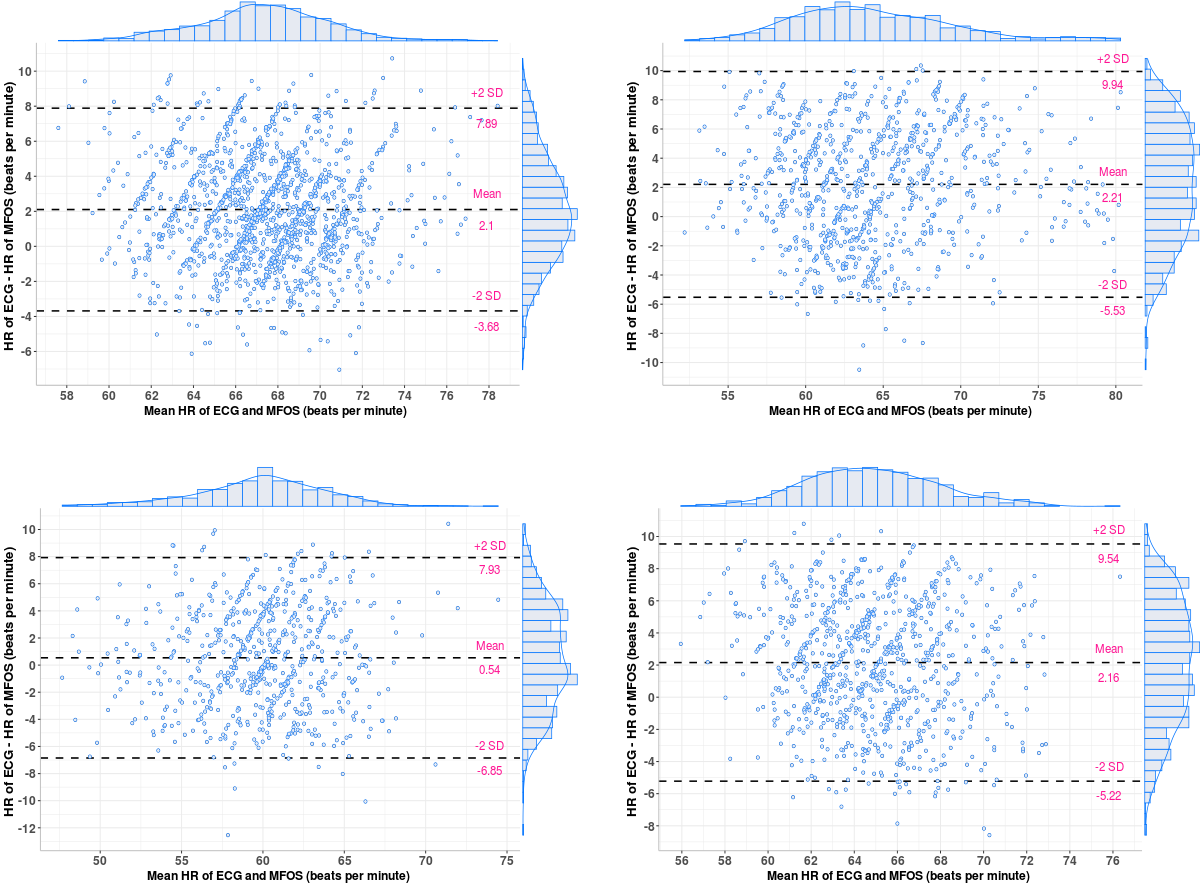
Bland-Altman plots of the heart rates for patients 1, 2, 6, and 10 (ie, top left, top right, bottom left, and bottom right, respectively). ECG: electrocardiogram; HR: heart rate; MFOS: microbend fiber optic sensor; RR: respiratory rate.

**Figure 10:**
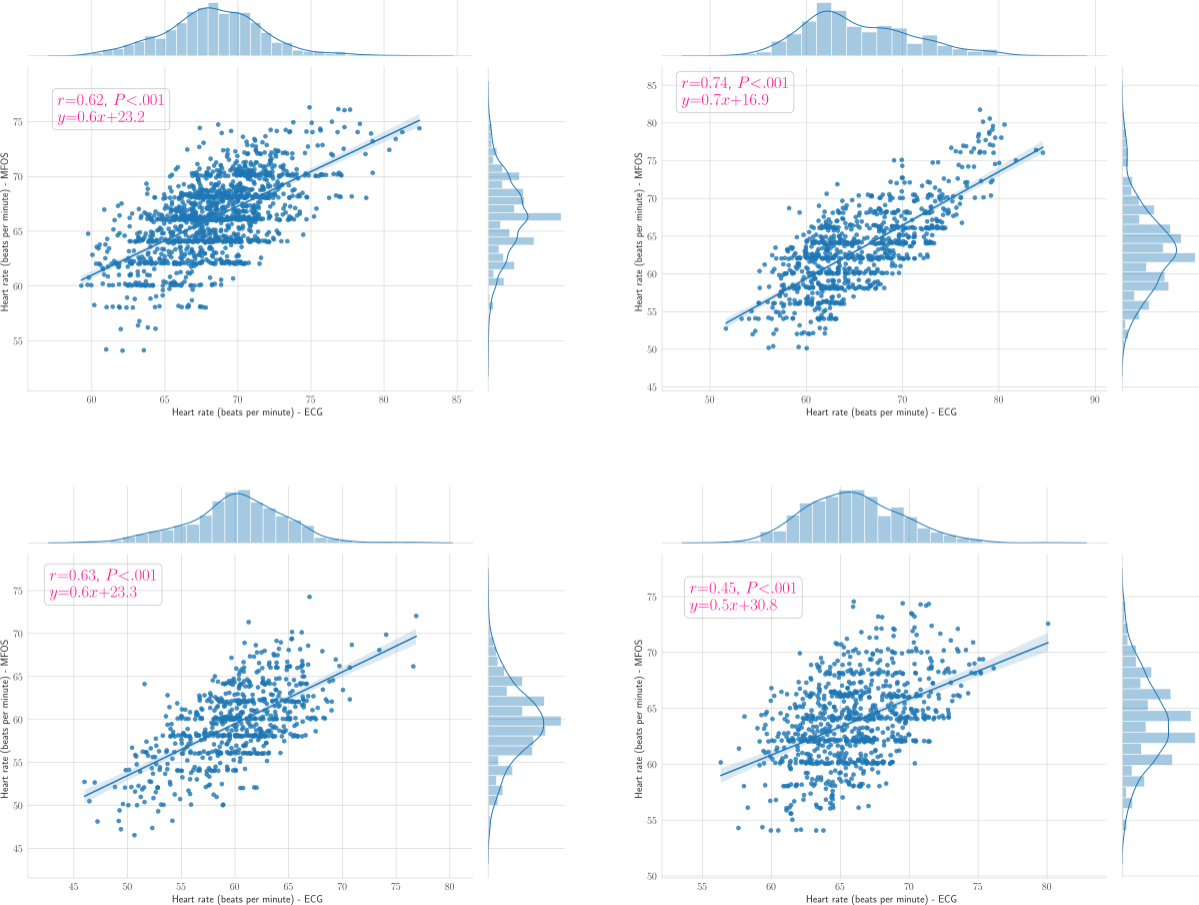
Pearson correlation plots of the heart rates for patients 1, 2, 6, and 10 (ie, top left, top right, bottom left, and bottom right, respectively). The blue circles represent reference heart rate against the estimated heart rate, and the blue line represents the fitted line. ECG: electrocardiogram; MFOS: microbend fiber optic sensor.

**Figure 11:**
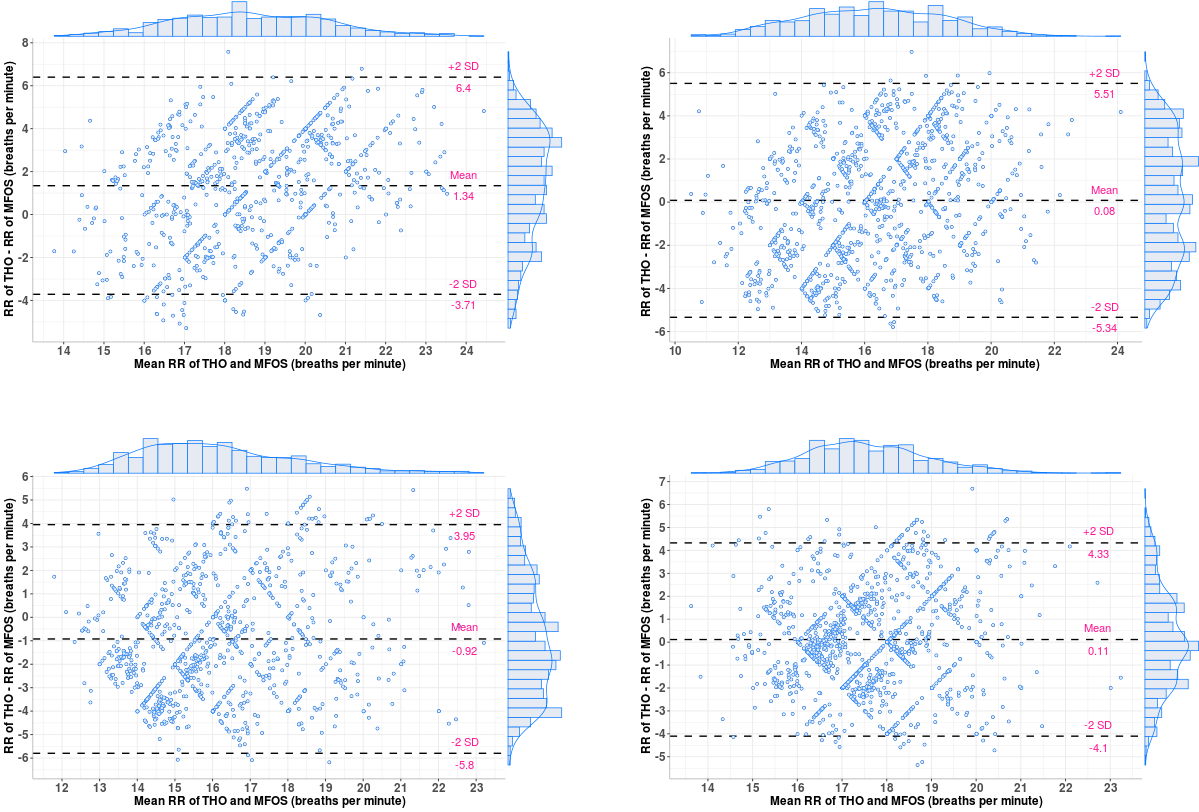
Bland-Altman plots of the respiratory rates for patients 3, 4, 5, and 9 (ie, top left, top right, bottom left, and bottom right, respectively). ECG: electrocardiogram; HR: heart rate; MFOS: microbend fiber optic sensor; RR: respiratory rate; THO: thoracic belt.

**Figure 12:**
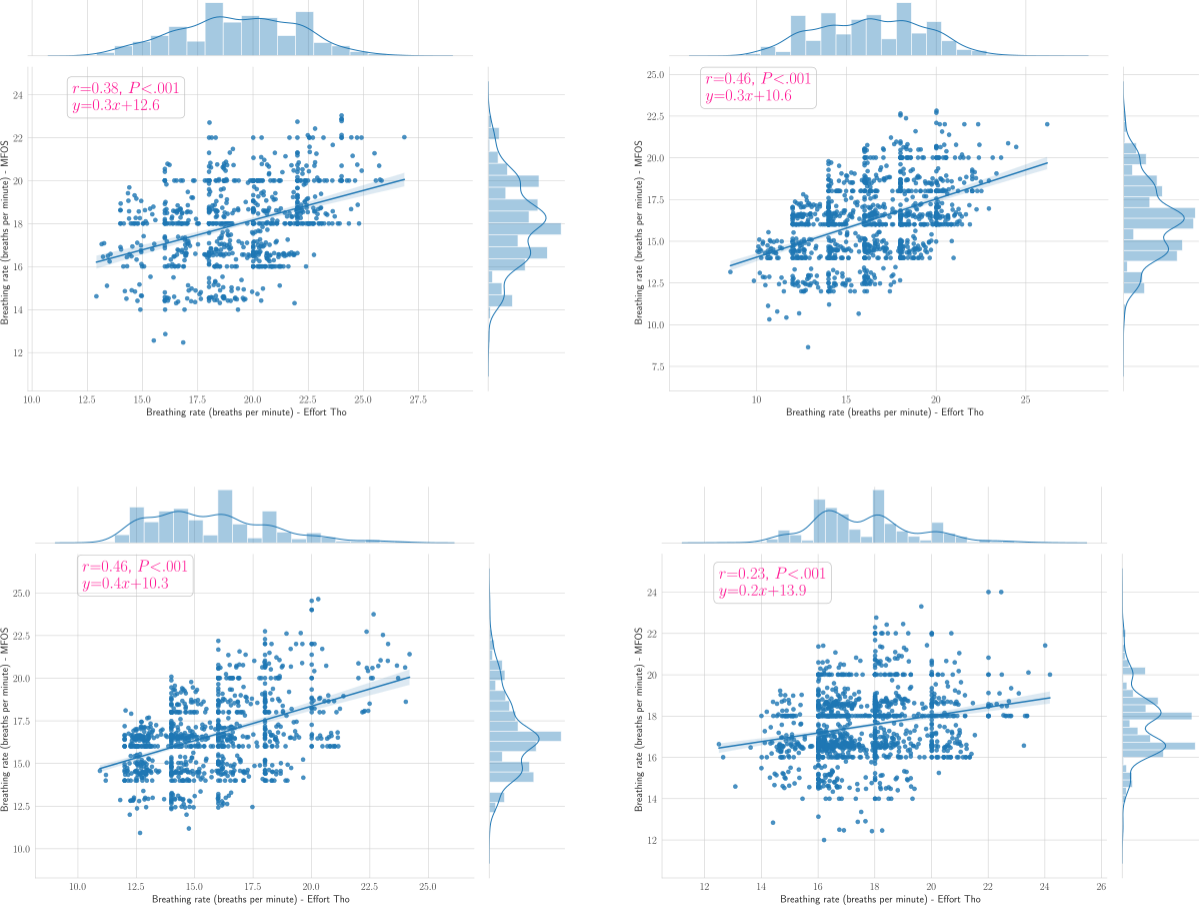
Pearson correlation plots of the respiratory rates for patients 3, 4, 5, and 9 (ie, top left, top right, bottom left, and bottom right, respectively). The blue circles represent reference RR against the estimated respiratory rate, and the blue line represents the fitted line. MFOS: microbend fiber optic sensor.

Tables 4 and 5 summarize the limits of agreement (LoA) of the Bland-Altman plot, *r* value, and *P* value for HRs and RRs, respectively. To provide some examples, we provided the Bland-Altman plots and Pearson correlation coefficient plots of the HRs for patients 1, 2, 6, and 10 in Figure 9 (top left, top right, bottom left, and bottom right, respectively) and Figure 10 (top left, top right, bottom left, and bottom right, respectively). In addition, we presented the Bland-Altman plots and the Pearson correlation coefficient plots of the BRs for patients 3, 4, 5, and 9 in Figure 11 (top left, top right, bottom left, and bottom right, respectively) and Figure 12 (top left, top right, bottom left, and bottom right, respectively).

**Table 4:** Limits of agreement of the Bland-Altman plots, Pearson correlation coefficient, and *P* value for heart rate detection.

Patients	Heart rate				
	Limits of agreement		SD of difference	*r*	*P* value
	Lower	Upper			
1	−3.68	7.89	2.95	0.62	<.001
2	−5.52	9.94	3.94	0.74	<.001
3	−9.46	7.41	4.30	0.68	<.001
4	−7.38	8.06	3.94	0.77	<.001
5	−5.80	8.59	3.67	0.31	<.001
6	−6.85	7.92	3.77	0.63	<.001
7	−8.68	6.44	3.86	0.70	<.001
8	−3.77	11.10	3.80	0.63	<.001
9	−7.13	8.28	3.93	0.64	<.001
10	−5.22	9.53	3.76	0.45	<.001

**Table 5:** Limits of agreement of the Bland-Altman plots, Pearson correlation coefficient, and *P* value for respiratory rate detection.

Patients	Respiratory rate							
	Limits of agreement			SD of difference		*r*		*P* value
	Lower	Upper						
1	−3.77	3.71	1.91		0.43		<.001	
2	−3.87	3.43	1.86		0.58		<.001	
3	−3.71	6.40	2.58		0.38		<.001	
4	−5.34	5.50	2.77		0.46		<.001	
5	−5.79	3.95	2.48		0.46		<.001	
6	−5.28	4.95	2.61		0.43		<.001	
7	−3.32	4.56	2.01		0.39		<.001	
8	−4.22	5.26	2.42		0.42		<.001	
9	−4.10	4.32	2.15		0.23		<.001	
10	−5.57	4.21	2.49		0.36		<.001	

### Apneic Event Detection

The apneic events were detected from the derived respiratory signals via windowing with overlapping. The assessment was made against the manually scored apneic events. Each sliding window was classified as either an apneic breathing event or a normal breathing event. Apneic events consisted of obstructive apneas, hypopnea, central apneas, and mixed apneas. For detection, we tested a sliding time window of 2 different sizes, that is, a sliding time window of 60 seconds with an overlap of 30 seconds as well as a sliding time window of 30 seconds with an overlap of 15 seconds. For the former, if any 20-second slice satisfied the apneic threshold condition, we considered the entire 60-second window as an apneic event. Similarly, for the latter, if any 10-second slice satisfied the apneic threshold condition, we considered the complete 30-second window as an apneic condition.

**Figure 13:**
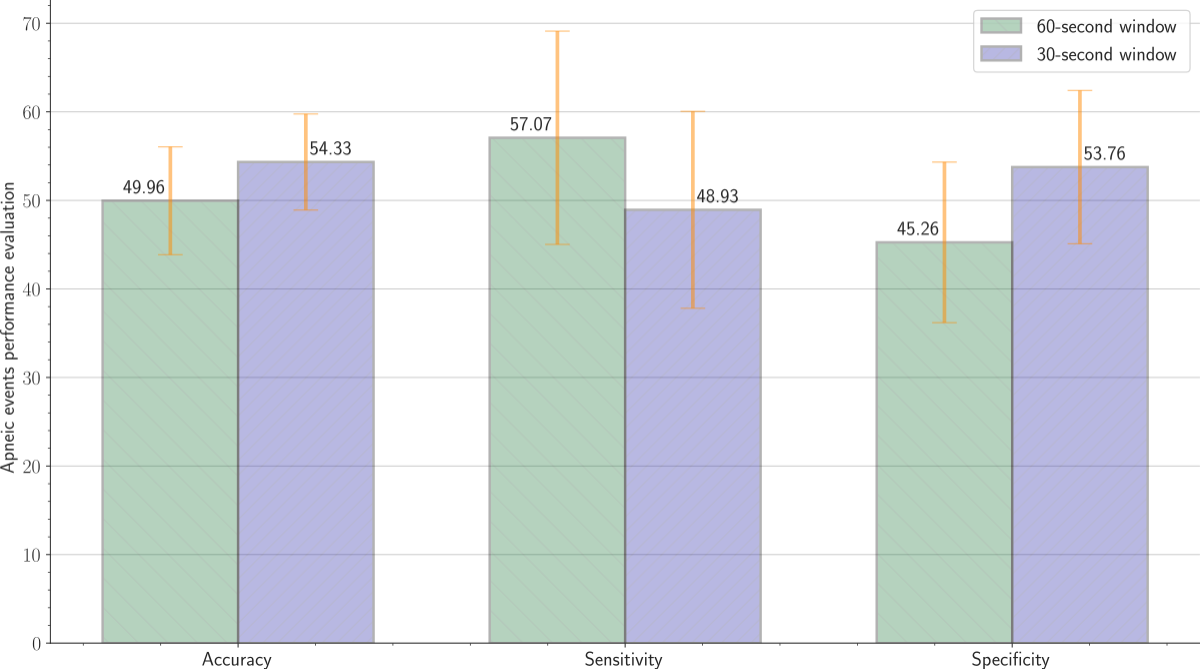
Bar charts with error bars for accuracy, sensitivity, and specificity regarding apneic events detection (60-second and 30-second time windows). The bars represent the mean of each measure, and the cap-tipped lines represent the uncertainty (SD) in each measure.

As presented in Table 6, for the 60-second time window, on average, the proposed system achieved an accuracy of 49.96% (SD 6.39), a sensitivity of 57.07% (SD 12.63), and specificity of 45.26% (SD 9.51). In addition, for the 30-second time window, on average, the proposed system achieved an accuracy of 54.33% (SD 5.72), a sensitivity of 48.93% (SD 11.72), and a specificity of 53.76% (SD 9.12). Figure 13 displays bar charts with error bars for the reported accuracy, sensitivity, and specificity related to apneic events’ detection of the 60-second and 30-second time windows.

**Table 6:** Accuracy, sensitivity, specificity, and *P* value of apneic event detection; the two-tailed test was used to determine the *P* value.

Characteristics		Patients									
		1	2	3	4	5	6	7	8	9	10
**Sliding time window of 60 seconds**											
	Accuracy (%)	49.95	53.93	39.26	46.29	46.53	63.2	57.01	50.8	46.56	46.02
	Sensitivity (%)	71.57	62.02	51.94	45.6	46.55	34.72	78.48	62.4	65.82	51.61
	Specificity (%)	45.24	52.26	37.56	46.42	46.52	69.54	33.9	38.86	41.28	41
	*P* value	<.001	<.001	<.001	<.001	<.001	<.001	<.001	.03	<.001	<.001
**Sliding time window of 30 seconds**											
	Accuracy (%)	55.18	58.05	47.19	51.05	56.35	66.82	56.88	53.13	49.24	49.44
	Sensitivity (%)	56.64	49.99	46.3	40.98	32.19	31.39	69.44	56.77	54.39	51.24
	Specificity (%)	54.86	59.7	47.31	52.94	59.62	74.71	43.38	49.39	47.83	47.83
	*P* value	<.001	<.001	<.001	<.001	<.001	<.001	<.001	<.001	<.001	.12

## Discussion

### Principal Findings

**Figure 14:**
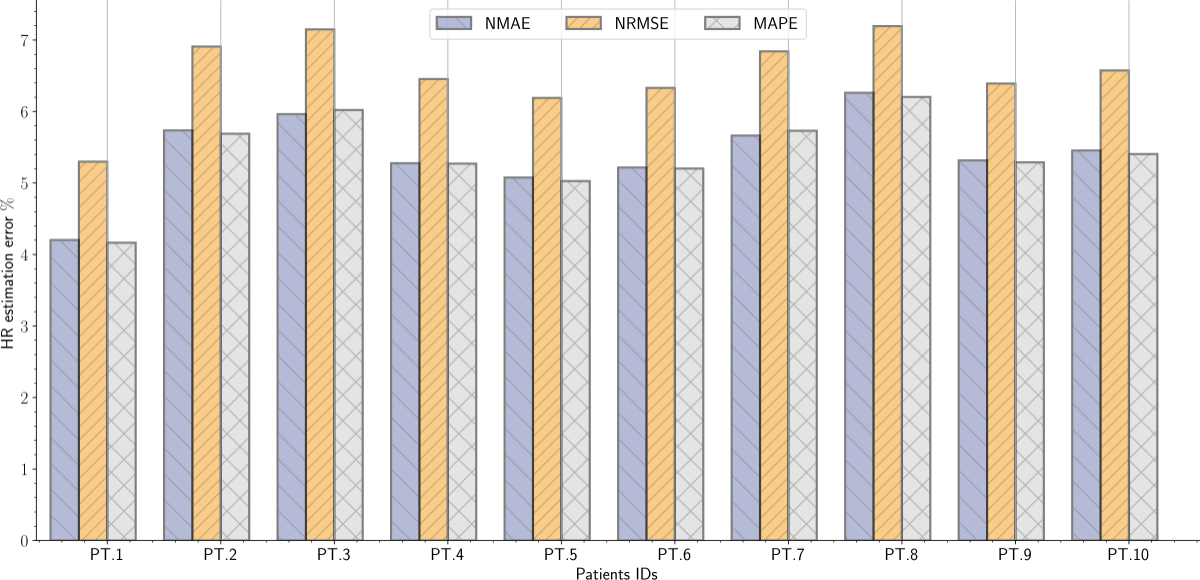
Bar plots of the normalized mean absolute error, normalized root mean square error, and mean absolute percentage error between reference device (electrocardiogram) and the microbend fiber optic sensor for heart rate estimation. HR: heart rate; MAPE: mean absolute percentage error; NMAE: normalized mean absolute error; NRMSE: normalized root mean square error.

In this study, we aimed to estimate the potential of using a single-channel monitoring device (ie, a bed-embedded FOS) for contactless monitoring of vital signs (ie, HRs and RRs) and apneic breathing events during an overnight sleep study. For HR estimation, the devised method achieved reasonably accurate results compared with the reference ECG signals. For the first patient, the system achieved the lowest NMAE, NRMSE, and MAPE, such as 4.20%, 5.30%, and 4.16%, respectively, whereas the highest NMAE, NRMSE, and MAPE were 6.26%, 7.19%, and 6.20%, respectively, for the eighth patient (Figure 14). The signal coverage for patients with severe OSA (eg, patients 8 and 10) was small compared with patients with moderate OSA (eg, patients 5 and 6). The signal coverage was the lowest for the eighth patient (ie, 51.24%), and the error in beats per minute was the highest among other patients. It is not necessarily true that patients with higher signal coverage will have the lowest error; however, the signal quality is the main factor affecting the outcomes. For instance, the first patient did not have the highest signal coverage (79.79%) but had the lowest error. This situation occurred because this patient had a small number of apneas (ie, 14) and a large number of hypopneas (ie, 191). The fifth patient had the highest coverage (87.51%); however, the error was slightly larger compared with the first patient. This situation occurred because this patient had a large number of apneas (ie, 203) and a small number of hypopneas (ie, 20). Overall, it may be said that the error in beats per minute is likely to increase for patients with a large number of apneas. This is because the amount of motion artifacts progresses for patients with severe apnea. In addition, the morphology of the BCG is significantly affected by cardiovascular complications of sleep apnea. It should also be recalled that the designated threshold value to eliminate motion artifacts had an effect on the estimation process. To explain, in our method, we rejected time windows that had an SD value 4 times greater than the MAD. Decreasing the threshold value would have allowed us to reject a large number of motion artifacts and consequently obtain lower errors. Nonetheless, the signal coverage could have been much lower. As a result, we balanced between achieving reasonable errors and retaining reasonable signal coverage. HR results were also supported by the Pearson correlation coefficients and LoA of the Bland-Altman plot. The system achieved the highest correlation coefficient for the fourth patient (*r*=0.77; *P*<.001) and the lowest correlation for the fifth patient (*r*=0.31; *P*<.001).

**Figure 15:**
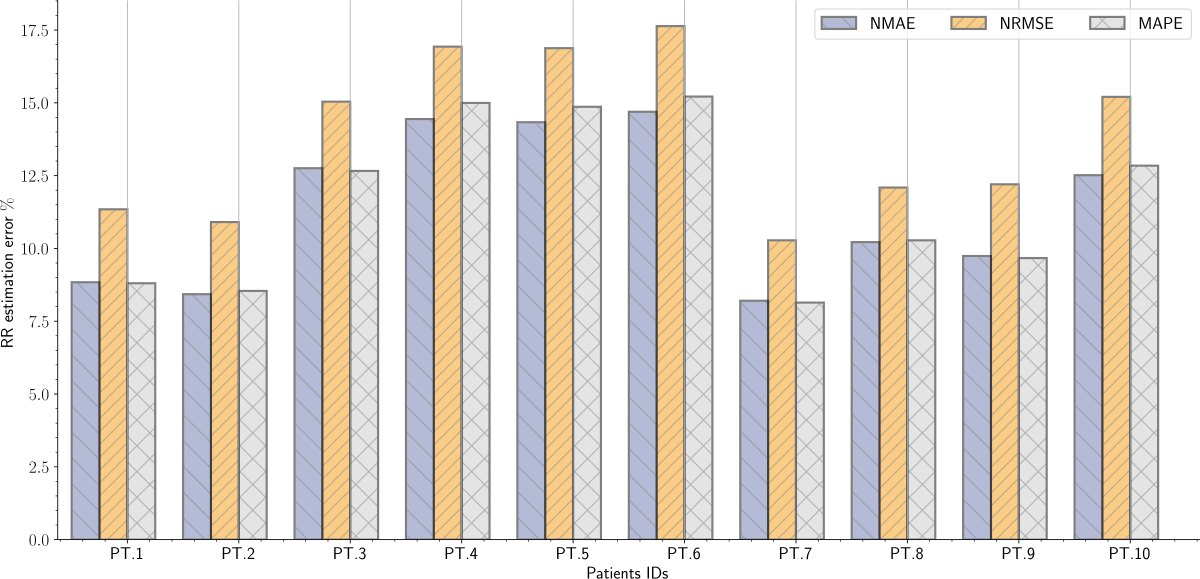
Bar plots of the normalized mean absolute error, normalized root mean square error, and mean absolute percentage error between reference device (thoracic belt) and the microbend fiber optic sensor for respiratory rate estimation. MAPE: mean absolute percentage error; NMAE: normalized mean absolute error; NRMSE: normalized root mean square error; RR: respiratory rate.

RR findings, on the other hand, were slightly inferior to HR results. By way of illustration, the lowest NMAE, NRMSE, and MAPE were 8.20%, 10.28%, and 8.14%, respectively, for the seventh patient, whereas the highest NMAE, NRMSE, and MAPE were 14.69%, 17.64%, and 15.22%, respectively, for the sixth patient (Figure 15). In general, detecting RRs in healthy subjects is simpler than detecting HRs. This is because respiratory cycles, that is, inhalation and exhalation, can be located through a peak detector. However, the situation is more challenging for patients with sleep apnea for different reasons. To illustrate, in our approach, RRs represent the movement of the chest and abdominal wall; however, because of the recurrent decrease and increase in breathing effort, detecting respiratory cycles has become a challenging task. These variations in breathing efforts affected the accuracy of the peak detector and consequently contributed to increasing the error between the devised sensor and the reference thoracic belt. Compared with HR detection, the lowest correlation coefficient was (*r*=0.23; *P*<.001) for the ninth patient, whereas the highest correlation was (*r*=0.58; *P*<.001) for the second patient. In general, patients with very severe OSA (ie, patients 7, 8, and 10) presented slightly worse correlation coefficients than patients with less severe OSA (ie, patients 1, 2, 4, 5, and 6). The value of the correlation coefficient depended to no small extent on the types of apneas and also the duration of apneic events presented in each patient. These parameters significantly influenced the respiratory signal’s typical shape, and thus, the respiratory cycles were more difficult to detect.

**Figure 16:**
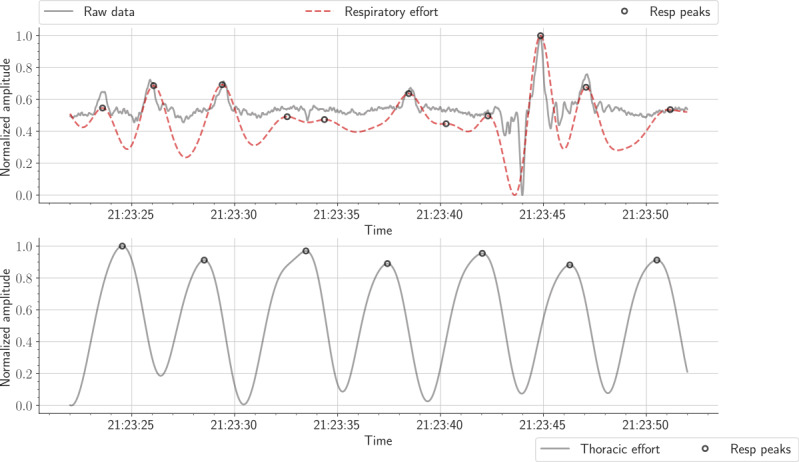
The first row displays a 30-second raw signal plus the respiratory effort signal for patient 6. The second row shows the equivalent thoracic belt signal.

**Figure 17:**
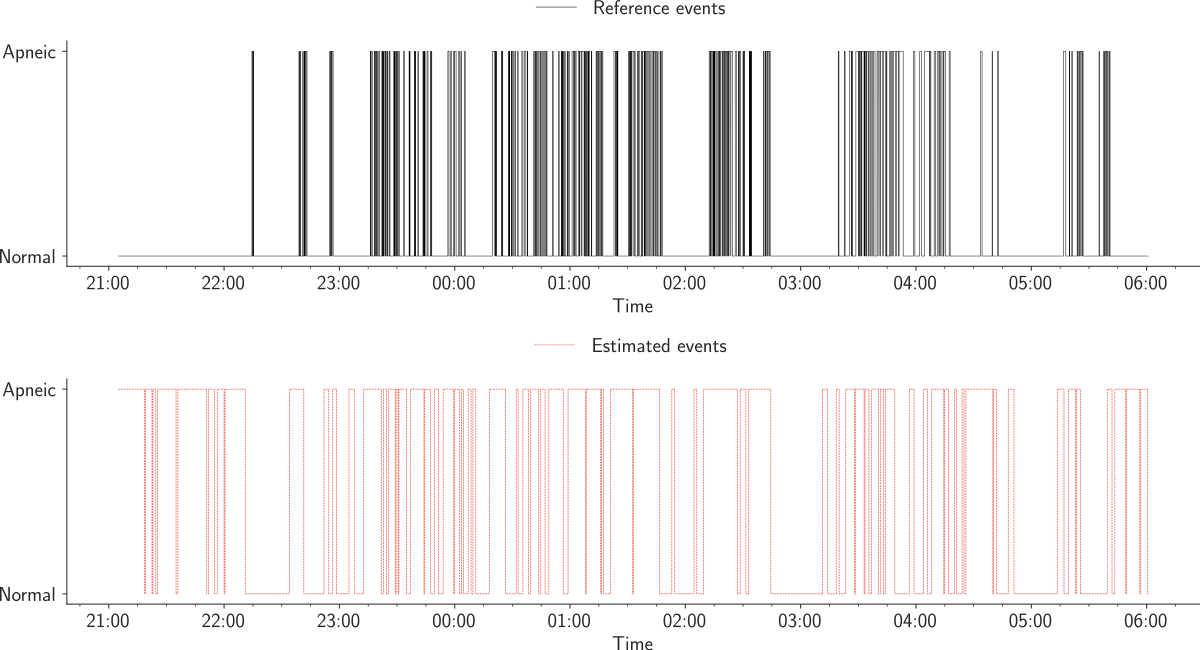
Annotation of apneic events for the first patient. The first row describes the reference events (apnea-hypopnea index is 36.8 events per hour), whereas the second row describes the estimated events (apnea-hypopnea index is 23 events per hour).

This highest error occurred in the sixth patient, most likely because of poor contact between the sensor mat and patient. The structure of both BCG and respiratory signals was highly different compared with that of other patients. Furthermore, the amplitude of the acquired raw data was very low. These issues contributed to a large discrepancy between the true peaks and detected peaks, as presented in Figure 16. Sleep apnea detection, from a different angle, demands multiple sensors and wires fixed to the patient’s body throughout one night, including, for example, airflow, respiratory effort, and oximetry; notwithstanding, in this study, we only employed a single-channel BCG sensor. The conceived sensor delivered reasonably good results considering the fact that we were using a single-channel BCG sensor. The detection evaluation metrics (ie, accuracy, sensitivity, and specificity) were measured according to the overlapping between manually scored events and events obtained by the deployed sensor mat. Across all recruited patients, the 60-second sliding moving window slightly outperformed the 30-second moving window. As shown in Table 6, the average sensitivity of the former window was 57.07% (SD 12.63) compared with 48.93% (SD 11.72) for the second window. Although the accuracy and specificity of the 30-second window were slightly better than those of the 60-second window, the *P* value of the last patient was .12. In contrast, the 60-second window reached a *P* value of <.001 for all patients but the eighth patient (*P*=.03). In addition, the lowest sensitivity for the 30-second window was 31.39%; however, it was 34.72% for the 60-second window. Both time windows achieved the highest sensitivity for the seventh patient, such as 78.48% and 69.44% in a row. Figure 17 presents an example of an apneic event annotation related to the first patient throughout the night.

Owing to the reasons mentioned earlier, the 60-second window was selected for apneic event detection. As discussed in the Parameter Selection section, a threshold of 0.45 was selected because it contributed to a balanced result between sensitivity and specificity. Selecting a smaller threshold value (eg, 0.2) would have resulted in a very high sensitivity across all patients; however, the opposite would have happened for both specificity and accuracy. Regarding the 0.45 threshold, the sensitivity tended to increase exponentially for patients with less severe OSA (ie, patient 5: AHI=26; patient 3: AHI=32.8; patient 2: AHI=33.7; and patient 1: AHI=36.8) in the order of 46.55%, 51.94%, 62.02%, and 71.57%, respectively. Nonetheless, the sixth patient (AHI=29 and sensitivity=34.72%) did not follow this order because of the presence of central apnea events (ie, 13 events). These events were more challenging to detect. On the other hand, the sensitivity tended to decrease exponentially in patients with very severe OSA (ie, patient 7: AHI=76.6%; patient 8: AHI=78.2; and patient 10: AHI=93.2) in the order of 78.48%, 62.40%, and 51.61%, respectively. This particular behavior was because of the apnea/hypopnea ratio. In other words, patients with a large number of hypopneas tended to have higher sensitivity when compared with other patients (Table 7). The designated threshold undoubtedly contributed to this outcome, and we could say that the proposed system was more appropriate for patients with less severe OSA. Such a configuration can be helpful in detecting OSA early and avoiding further complications. Table 7 presents the number of apneic events (obstructive apneas, hypopneas, central apneas, and mixed apneas) for each patient versus the proposed system’s sensitivity and the manually scored AHI.

**Table 7:** The counts of the different apneic events for each patient versus achieved sensitivity and the manually scored apnea-hypopnea index.

Patients	Obstructive apneas (n)	Hypopneas (n)	Central apneas	Mixed apneas	Sensitivity	Apnea-hypopnea index
1	12	189	0	0	71.57	36.8
2	23	168	13	0	62.02	33.7
3	11	92	0	0	51.94	32.8
4	201	18	0	0	45.6	58.3
5	47	50	0	0	46.55	26
6	90	64	13	0	34.72	29
7	499	47	1	9	78.48	76.6
8	394	21	3	34	62.4	78.2
9	132	127	1	0	65.82	54.8
10	577	24	2	0	51.61	93.2

### Limitations

The limitation of this study is the small sample size; despite that, our ultimate goal was to quantify the predictive outcomes of the fiber optic mat for vital signs and sleep apnea detection in a real-life sleep study. For HR and RR, the findings of the study have shown that the proposed system can provide results close to those of reference devices used in the PSG study. For sleep apnea detection, the designed system provided favorable results for patients with less severe OSA compared with patients with very severe OSA. This issue can be investigated in the future by adding another sensor, for example, an accelerometer, as a noise reference to eliminate body movements [49]. It should be pointed out that the suggested method for apnea detection did not follow the supervised learning models, and hence, we avoided labeling sensor data. The manually scored apneic events could have been used as a guide to label sensor data; however, the labeling process will be a restricted property, given large-scale deployment at users’ homes. Another issue to consider is data availability; BCG signals are not benchmarked; as a result, a training model can only be limited to specific sensor data. This problem occurs because the outcome of BCG sensors is not necessarily similar, which restricts testing across different data sets. As stated by Inan et al [18] in a recent review article, there should be a comprehensive and open database of BCG signals. Such databases will allow researchers to employ them in their environments and improve the field into an accepted technique appropriate for clinical studies [18].

### Comparison With Prior Work

We attempted to detect apneic events in a previous study [41], where the trial was performed during a drug-induced sleep endoscopy, and the optical fiber mat was compared with the ApneaLink device (ResMed). In a previous clinical study, the system delivered very low sensitivity because of the short evaluation period, that is, around 120 min per study. In addition, the ApneaLink device is not as accurate as the gold-standard PSG. Moreover, the employed algorithm did not consider the fact that there will always be a significant variation in the signal amplitude because of the chest movement. Therefore, a smaller threshold was selected to achieve realistic results. In this study, to mitigate these issues, the analysis was completed during a realistic overnight sleep study with the PSG as a gold standard for comparison. In addition, we improved the apneic detection algorithm to cope with real-life scenarios.

### Conclusions

In this study, we evaluated a single-channel monitoring device for detecting vital signs, namely, HR and RR, as well as sleep apnea events. The monitoring device consisted of a mat embedded with a microbending multimode fiber. We consolidated data from 10 patients diagnosed with OSA, in which the devised sensor mat was placed underneath the patient’s mattress, and raw data were collected without altering any typical configuration for the overnight sleep study. A wavelet-based analysis method was implemented for HR estimation, and satisfactory results were obtained in comparison with the reference ECG. RRs were detected from the derived effort respiratory signal after removing the nonlinear trend. Furthermore, the proposed method delivered results close to those of the reference piezoelectric thoracic belt. Both HR and RR were computed via a sliding time window of 30 seconds with an overlap of 15 seconds. The apneic events were detected on a minute-by-minute basis through an adaptive histogram-based thresholding approach. The suggested method provided average results for the distinction between normal breathing and apneic breathing events. Nevertheless, the results are encouraging considering the relative complexity of diagnosing sleep apnea via PSG. Indeed, the proposed sensor is not designed to substitute the gold-standard method. However, it can be seen as an assistive tool capable of providing longitudinal data without interfering with the subject’s everyday activities. Longitudinal data enable monitoring trends in vital signs that, in turn, can help to predict clinical deterioration in patients diagnosed with sleep-disordered breathing or cardiovascular diseases. In future work, we plan to integrate pulse oximetry with the proposed sensor mat to investigate the impact of adding another sensing modality for apneic event detection.

## Appendix

Multimedia Appendix 1 Additional information about the MFOS, data analysis, vital signs detection, as well as statistical analysis.

Multimedia Appendix 2 High-quality PNG images.

## Abbreviations

AAD: average absolute deviation
AHI: apnea-hypopnea index
BCG: ballistocardiogram
BR: breathing rate
ECG: electrocardiogram
EMD: empirical mode decomposition
HR: heart rate
HSAT: home sleep apnea test
LoA: limits of agreement
LOOCV: leave-one-out-cross-validation
MAD: median absolute deviation
MAPE: mean absolute percentage error
MFOS: microbend fiber optic sensor
MODWT: maximal overlap discrete wavelet transform
NHG: National Healthcare Group
NMAE: normalized mean absolute error
NRMSE: normalized root mean square error
OSA: obstructive sleep apnea
PSG: polysomnography
RR: respiratory rate
