# Supplementary material for: A New Approach for Detecting Sleep Apnea Using a Contactless Bed Sensor: Comparison Study
Source: J Med Internet Res. 2020 Sep 18;22(9):e18297. doi: 10.2196/18297 (PMC7532465; doi:10.2196/18297)
Supplement: Multimedia Appendix 1 [file jmir_v22i9e18297_app1.pdf]

Supplementary Material 1

Microbend Fiber-optic sensor

If the waveguide of a multimode fiber is subjected to mechanical perturbations, a redistribution of the light power among the several modes in the fiber occurs. The severity of such perturbations defines how much light is coupled to radiation modes [1,2]. For that reason, if we apply pressure to a squeezed graded-index multimode fiber between two layers of grating structures (i.e., tooth blocks), we can produce mechanical perturbations in the fiber, in our case, the sensor mat comprises a 10-meter loop of multimode fiber. The applied pressure allows coupling the transmission modes in the multimode fiber into the loss mode. This coupling reduces the amount of light received by a photodetector. Next, the photodetector converts the light signal into an electrical current signal. Then, a trans-impedance amplifier converts the current signal into a voltage signal. At last, the voltage signal is filtered by a 20 Hz low-pass filter and digitized by a 16-bit analog-to-digital converter [3].

Data Analysis

For each patient, encrypted binary files were first decrypted using proprietary software and stored in comma-separated values (CSV) file format. Afterward, all CSV files were concatenated into a single CSV file representing a one-night data recording. Each CSV file contained seven data columns, i.e., Unix timestamp, amplified raw data (1e7 x electric current), filtered BCG signal, ambient sound, ambient temperature, ambient light, unamplified raw data (1e6 x electric current), and the power supplied to the light source. We only considered the first and the seventh data columns in our data analysis, i.e., Unix timestamp and unamplified raw data. Upon binding data chunks for each patient, we synchronized acquired raw data according to the start and end time of the PSG study. Essentially, the unamplified raw data represent a mixture of two signals (i.e., BCG and respiratory effort signals), in addition to noise data or motion artifacts that represent frequent body movements.

Vital signs detection

Chebyshev Type I bandpass filter was applied to artifact-free data to obtain BCG and respiratory signals. The cutoff frequencies were selected such as (2.5Hz - 5Hz, 0.5dB) and (0.01Hz - 0.4Hz, 0.5dB), respectively. Several attempts in literature were made to compute the heart rate from BCG signals. These attempts include time-domain approaches, frequency-domain approaches, wavelet analysis, and clustering-based approaches [4]. A recent comparative study performed by Suliman et al. [4] concluded that the wavelet analysis-based approach proposed by Sadek et al. [5] was one of the two-high performing methods in terms of average peak detection rate, average false alarm rate, and average mean absolute error between true and predicted peaks. This particular task is challenging because the J-peaks of the BCG signal (equivalent to R-peaks of the ECG signal) are not consistent and vary across and between subjects. For J-peak detection, we used Sadek et al. [5] approach, which utilizes the multiresolution analysis of the "maximal overlap discrete wavelet transform", or a.k.a., MODWT [6]. This method aimed at reducing the BCG signal into smooth and detail time series components by passing the signal through low-pass and high-pass filters and then selecting the component that shows an agreement with the J-peaks. The Biorthogonal wavelet Bior3.9 basis function with level 4 was accommodated for the analysis, while the fourth level smooth coefficient was chosen to represent the cardiac cycles. In the end, J-peaks were traced

through a peak detector. The same wavelet basis function was used across all patients recruited in the study. Heart rates were measured using a sliding time window of 30 seconds with an overlap of 15 seconds. The ECG signal was used as a reference to detect interbeat intervals (IBIs). For this purpose, we selected the well-known Pan and Tompkins algorithm owing to its reasonable results [7]. Respiratory rate, on the other hand, can be measured directly from the band passed filtered data via a peak detector. However, before locating breathing cycles, we first removed the nonlinear trend from the signal by subtracting a polynomial fit of the 3rd order. Respiratory rates were calculated using a sliding time window of 30 seconds with an overlap of 15 seconds. The effort signal obtained from the thoracic belt was used as a reference to detect respiratory cycles. Compared to abdominal effort and airflow (i.e., pressure and thermistor) signals, effort thoracic signal was highly correlated with the one acquired from the optical fiber mat.

Statistical analysis

All data processing and analysis were presented in Python (version 3.7.6) using PyCharm Professional Edition. Graphical illustrations of data analysis and evaluation metrics, including, for example, the Pearson correlation coefficient and bar plots with error bars were produced by python. Seaborn 0.10.0 Python data visualization library was used to create the Pearson correlation coefficient plots, while RStudio version 1.2.5033 (Rstudio Inc) was used to create the Bland-Altman plots. Table 1 shows the error metrics used in our approach along with their mathematical formulas.

Table 1 Error metrics employed in our analysis along with their mathematical formulas.

Error Metric	Formula and definitions
**Sensitivity**	• $Sens = \frac{TP}{TP+FP}$<br>○ TP is the number of events correctly identified as apneas.<br>○ FP is the number of events incorrectly identified as apneas.
**Specificity**	• $Sepc = \frac{TN}{FP+TN}$<br>○ TN is the number of events correctly identified as non-apneas.
**Accuracy**	• $Acc = \frac{TP+TN}{TN+TN+FN+FN}$<br>○ FN is the number of events incorrectly identified as non-apneas.
**Cohen Kappa coefficient**	• $kappa = \frac{P_O - P_C}{1 - P_C}$<br>○ P_o is the relative observed agreement among readers.<br>○ P_c is the hypothetical probability of chance agreement.
**Matthews correlation coefficient**	• $MCC = \frac{TP \ x \ TN - FP \ x \ FN}{\sqrt{(TP+FP)(TP+FN)(TN+FP)(TN+FN)}}$
**Pearson correlation coefficient**	• $r_{xy} = \frac{n\sum x_i y_i - \sum x_i \sum y_i}{\sqrt{n\sum x_i^2 - (\sum x_i)^2}\sqrt{n\sum y_i^2 - (\sum y_i)^2}}$<br>○ n is number of observations<br>○ x_i is the value of x (for ith observation)<br>○ y_i is the value of y (for ith observation)
**Normalized root mean square error**	• $NRMSE = \frac{\sqrt{\frac{1}{n}\sum_{i=1}^{n}(\hat{y}_i - y_i)^2}}{\max(y) - \min(y)}$<br>○ $\hat{y}$ is the predicted value.<br>○ y is the true value.<br>○ μ is the mean of the observed values.
**Normalized mean absolute error**	• $NMAE = \frac{\frac{1}{n}\sum_{i=1}^{n}\lvert\hat{y}_i - y_i\rvert}{\max(y) - \min(y)}$

Sensitivity

Sensitivity (Sens) is often presented in proportion and describes the probability that a test will yield a positive result if the disorder is present. It is determined as the number of correct positive predictions divided by the total number of positives [8–10]. Likewise, it can be described as a recall or true positive rate. In our case, it defines the proportion of correctly identified apneic events.

Specificity

Specificity (Spec) is often presented in proportion and describes the probability that a test will yield a negative result if the disorder is not present. It is calculated as the number of correct negative predictions divided by the total number of negatives [8–10]. Likewise, it can be described as a true negative rate. In our case, it defines the proportion of correctly identified non-apneic events.

Accuracy

Accuracy (Acc) is often presented in proportion and describes the probability of all instances that are correctly classified. It is calculated as the number of all correct predictions divided by the total number of all instances in the dataset [8–10].

Cohen kappa coefficient

The Cohen kappa coefficient, i.e., kappa statistic, is often adopted to measure the inter-annotator agreement. In other words, it presents the percentage of agreement beyond that predicted by chance [11]. In common with the correlation coefficient, it can vary from -1 to 1, where 0 defines the amount of agreement that can be predicted from random chance, and 1 suggests a precise agreement between the raters. According to Cohen [12], we can translate kappa results as follows: "values ≤ 0 as indicative of no agreement and 0.01-0.20 as none to slight, 0.21-0.40 as fair, 0.41-0.60 as moderate, 0.61–0.80 as substantial, and 0.81–1.00 as an almost perfect agreement" [13].

Matthews correlation coefficient

Matthews correlation coefficient (MCC) is a special case of the Pearson correlation coefficient, i.e., it is a cross-tabulation method of calculating the Pearson correlation coefficient between true and predicted values. The value of the coefficient varies between -1 and +1. A coefficient of +1 denotes a perfect classification, 0 a coin-tossing classifier and -1 a perfect misclassification. It is also identified as the phi coefficient and the only binary classifier that can yield a high score only when the binary predictor could accurately predict the most positive data instances and most negative data instances [14].

Bland-Altman plot

The Bland-Altman plot [15,16] is a tool to quantify the agreement between two quantitative estimations. This is done by creating limits of agreement (LoA). The LoA are calculated using the mean and standard deviations of the differences between the two measurements [17]. This graphical representation plots the differences between the two measurements on the y-axis and the averages of the two measurements on the x-axis. It is a favored method to measure the agreement between two medical devices because devices are not likely to have the exact agreement. Most importantly, it estimates how close pairs of measurements are as small differences between devices are not likely to influence patient decisions [18].

Pearson correlation coefficient

Pearson correlation coefficient (r) measures the statistical relationship between two continuous variables. It can provide information about the magnitude and direction of the relationship. The value of the coefficient varies between -1 and +1. A perfect degree of association occurs if the value is ±1. The relationship between the two variables becomes weaker when the value of the coefficient goes toward 0. The sign of the coefficient indicates the direction of the relationship. A positive relationship occurs when the coefficient has a positive sign and a negative relationship occurs when the coefficient has a negative sign.

Normalized root mean square error

Root mean square error (RMSE) is an often-used estimator that measures the average of the squares of the errors. It varies from 0 to ∞ and does not consider the direction of the error. Normalized RMSE (NRMSE) is simply the RMSE normalized by the range of the observed data and can be explained as a fraction of the overall range that is typically resolved by the model.

Normalized mean absolute error

The mean absolute error (MAE) denotes the average distance between each data point and the mean. As the name suggests, it is the absolute value of the difference between forecasted values and actual values. It varies from 0 to ∞ and does not consider the direction of the error. Normalized MAE (NMAE) is simply the MAE normalized by the range of the observed data.

References

1. Lagakos N, Cole JH, Bucaro JA. Microbend fiber-optic sensor. Appl Opt. 1987;26(11):2171–80. PMID: 20489839
2. Luo F, Liu J, Ma N, Morse TF. Fiber optic microbend sensor for distributed sensing application in the structural strain monitoring. Sensors Actuators, A Phys. 1999;75(1):41–44.
3. Sadek Ibrahim Hussein Tahoun I. Nonintrusive Nocturnal Remote Monitoring of Vital Signs in Ambient Assisted Living Environments [Internet]. Université Montpellier; 2018.
4. Suliman A, Carlson C, Ade CJ, Warren S, Thompson DE. Performance Comparison for Ballistocardiogram Peak Detection Methods. IEEE Access. IEEE; 2019;7:53945–53955.
5. Sadek I, Biswas J, Abdulrazak B, Haihong Z, Mokhtari M. Continuous and unconstrained vital signs monitoring with ballistocardiogram sensors in headrest position. 2017 IEEE EMBS Int Conf Biomed Heal Informatics. IEEE; 2017. p. 289–292.
6. Percival DB, Walden AT. Wavelet Methods for Time Series Analysis [Internet]. Cambridge: Cambridge University Press; 2000.
7. Pan J, Tompkins WJ. A real-time QRS detection algorithm. IEEE Trans Biomed Eng. 1985;32(3):230–6. PMID: 3997178
8. Wong HB, Lim GH. Measures of Diagnostic Accuracy: Sensitivity, Specificity, PPV and NPV. Proc Singapore Healthc. 2011;20(4):316–318.
9. Eusebi P. Diagnostic accuracy measures. Cerebrovasc Dis. 2013;36(4):267–72. PMID: 24135733
10. Baratloo A, Hosseini M, Negida A, El Ashal G. Part 1: Simple Definition and Calculation of Accuracy, Sensitivity and Specificity. Emerg (Tehran, Iran). 2015;3(2):48–9. PMID: 26495380
11. Sim J, Wright CC. The Kappa Statistic in Reliability Studies: Use, Interpretation, and Sample Size Requirements. Phys Ther. 2005;85(3):257–268. PMID: 15733050
12. Cohen J. A Coefficient of Agreement for Nominal Scales. Educ Psychol Meas. 1960;20(1):37–46.
13. McHugh ML. Interrater reliability: the kappa statistic. Biochem medica. 2012;22(3):276–82. PMID: 23092060
14. Chicco D, Jurman G. The advantages of the Matthews correlation coefficient (MCC) over F1 score and accuracy in binary classification evaluation. BMC Genomics. BMC Genomics; 2020;21(1):6. PMID: 31898477
15. Bland JM, Altman DG. Statistical methods for assessing agreement between two methods of clinical measurement. Lancet (London, England). 1986;1(8476):307–10. PMID: 2868172
16. Bland JM, Altman DG. Measuring agreement in method comparison studies. Stat Methods Med Res. 1999;8(2):135–60. PMID: 10501650
17. Giavarina D. Understanding Bland Altman analysis. Biochem Medica. 2015;25(2):141–151. PMID: 26110027
18. Nelson BW, Allen NB. Accuracy of Consumer Wearable Heart Rate Measurement During an Ecologically Valid 24-Hour Period: Intraindividual Validation Study. JMIR mHealth uHealth. 2019;7(3):e10828. PMID: 30855232
