# Supplementary figures and images for: A New Approach for Detecting Sleep Apnea Using a Contactless Bed Sensor: Comparison Study
Source: J Med Internet Res. 2020 Sep 18;22(9):e18297. doi: 10.2196/18297 (PMC7532465; doi:10.2196/18297)

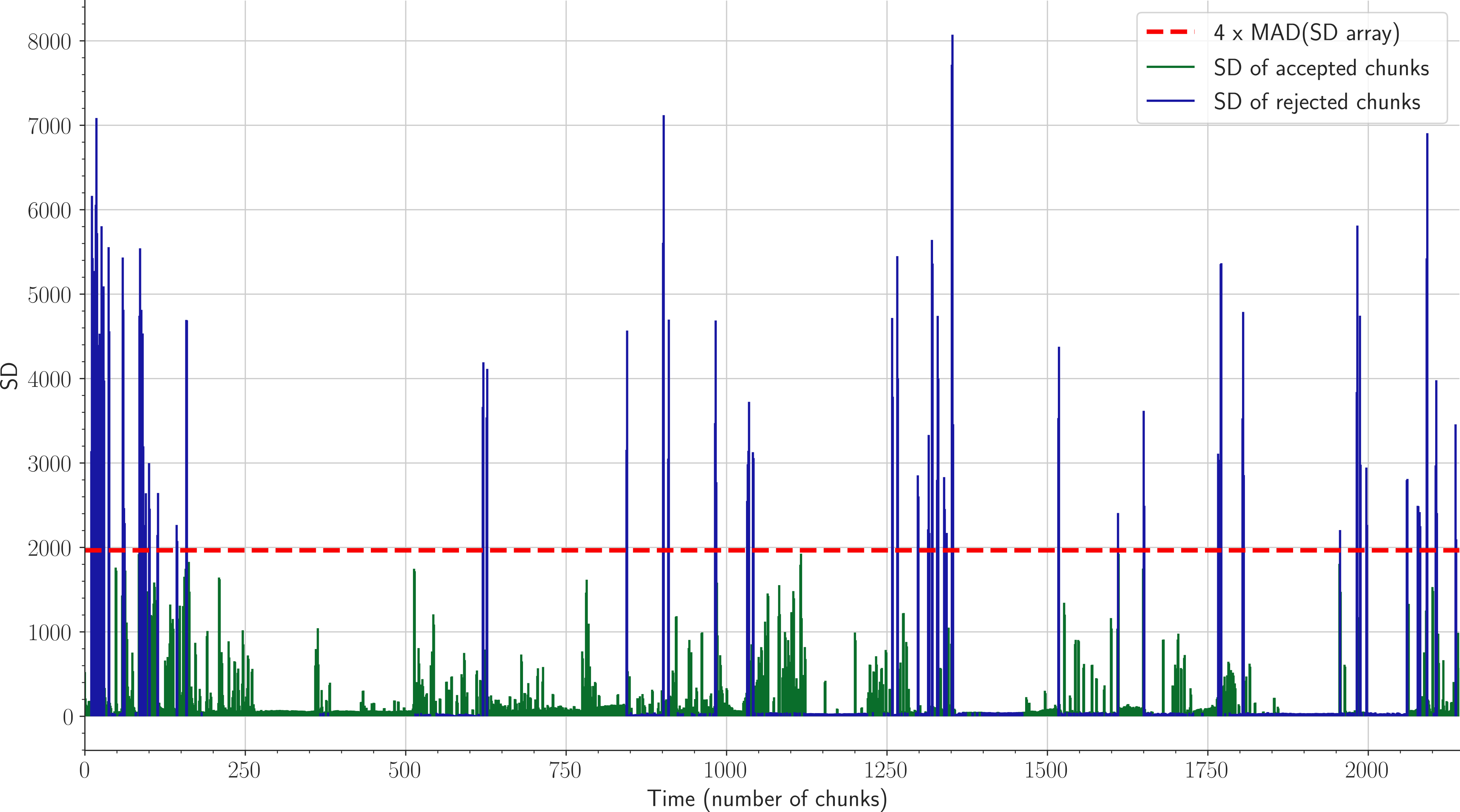

Supplement: Multimedia Appendix 2 [file jmir_v22i9e18297_app2.zip › high_quality_source_png_images_0002/Figure_0002.png]

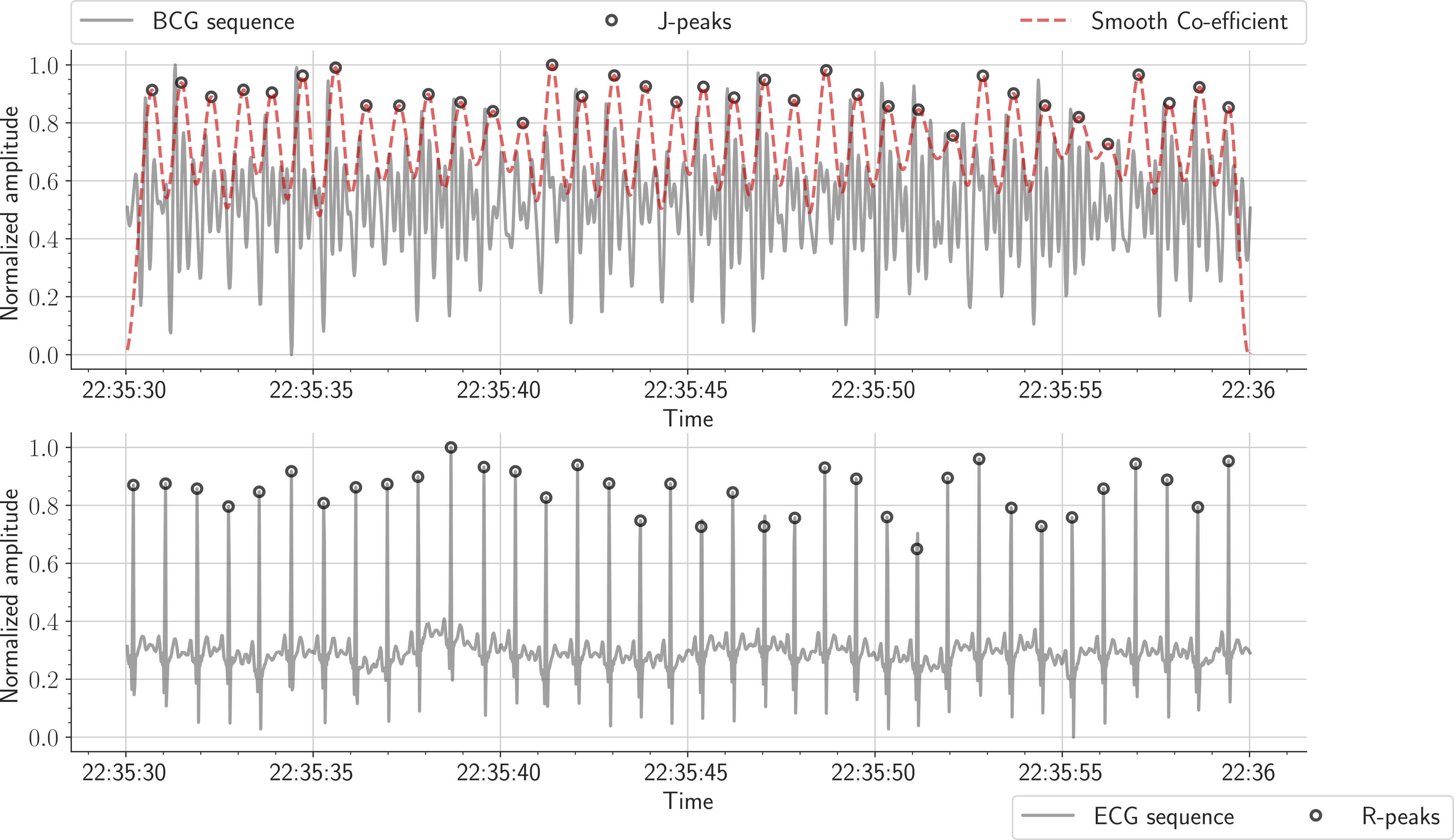

Supplement: Multimedia Appendix 2 [file jmir_v22i9e18297_app2.zip › high_quality_source_png_images_0002/Figure_0003.png]

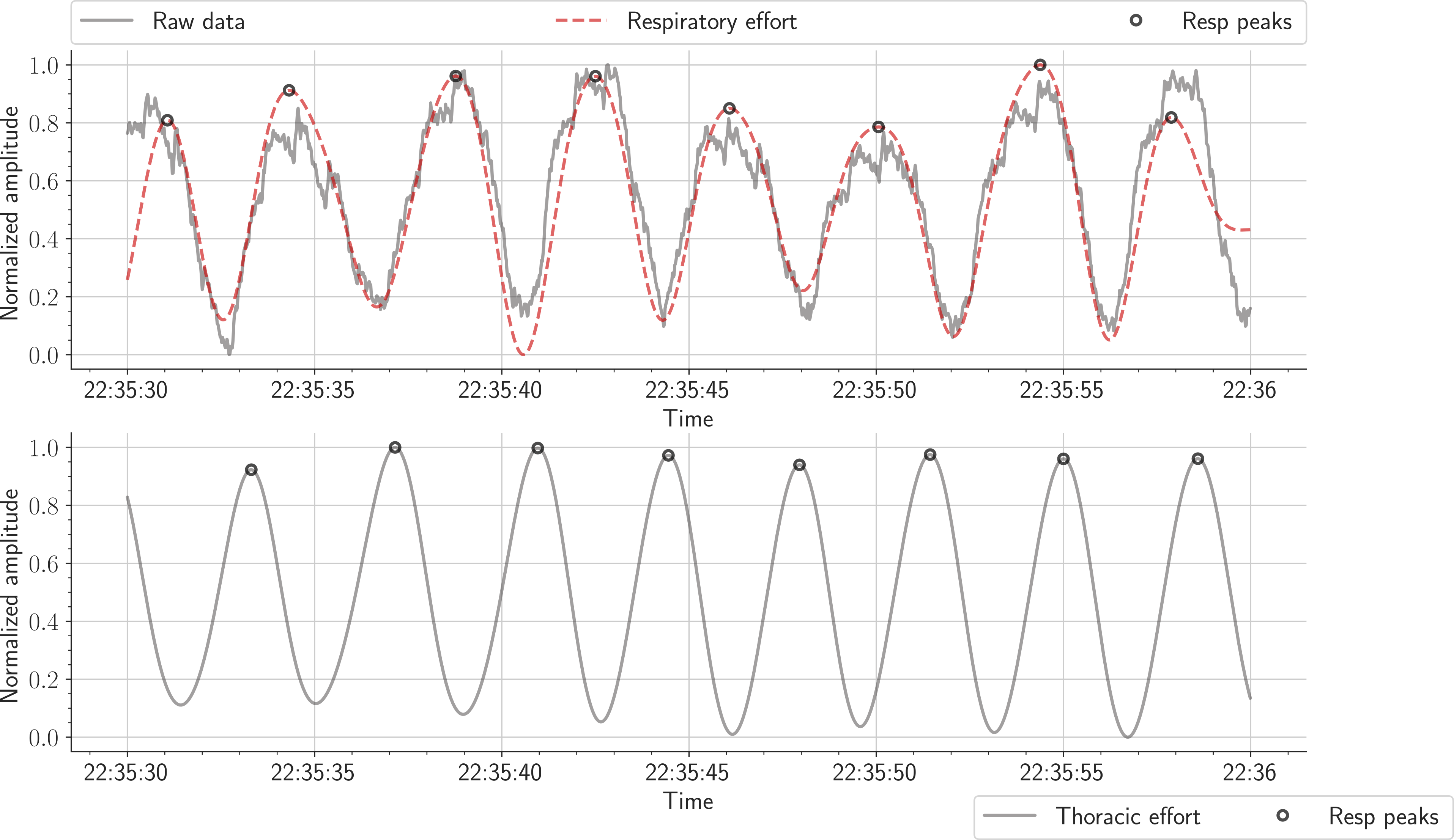

Supplement: Multimedia Appendix 2 [file jmir_v22i9e18297_app2.zip › high_quality_source_png_images_0002/Figure_0004.png]

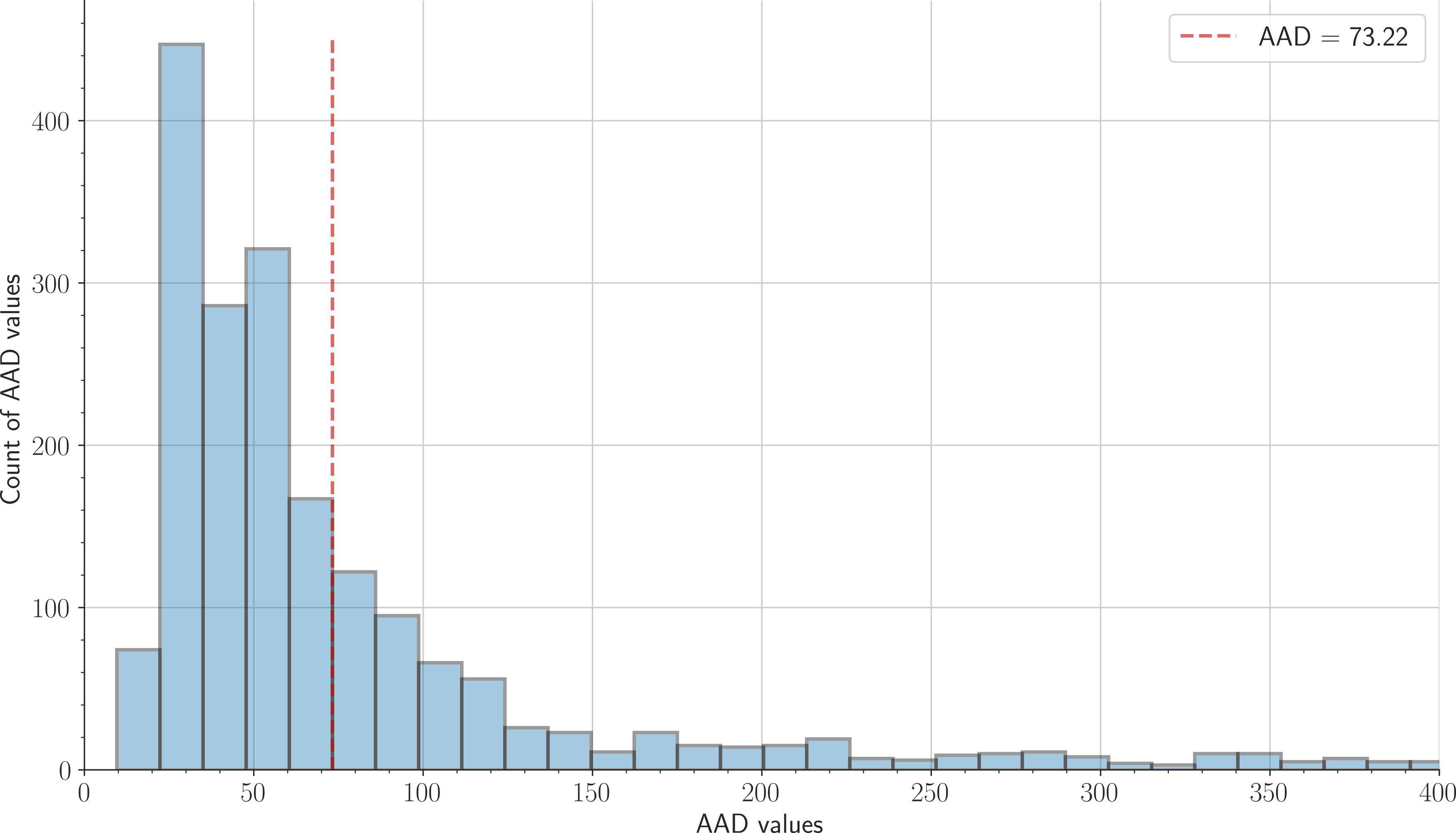

Supplement: Multimedia Appendix 2 [file jmir_v22i9e18297_app2.zip › high_quality_source_png_images_0002/Figure_0005.png]

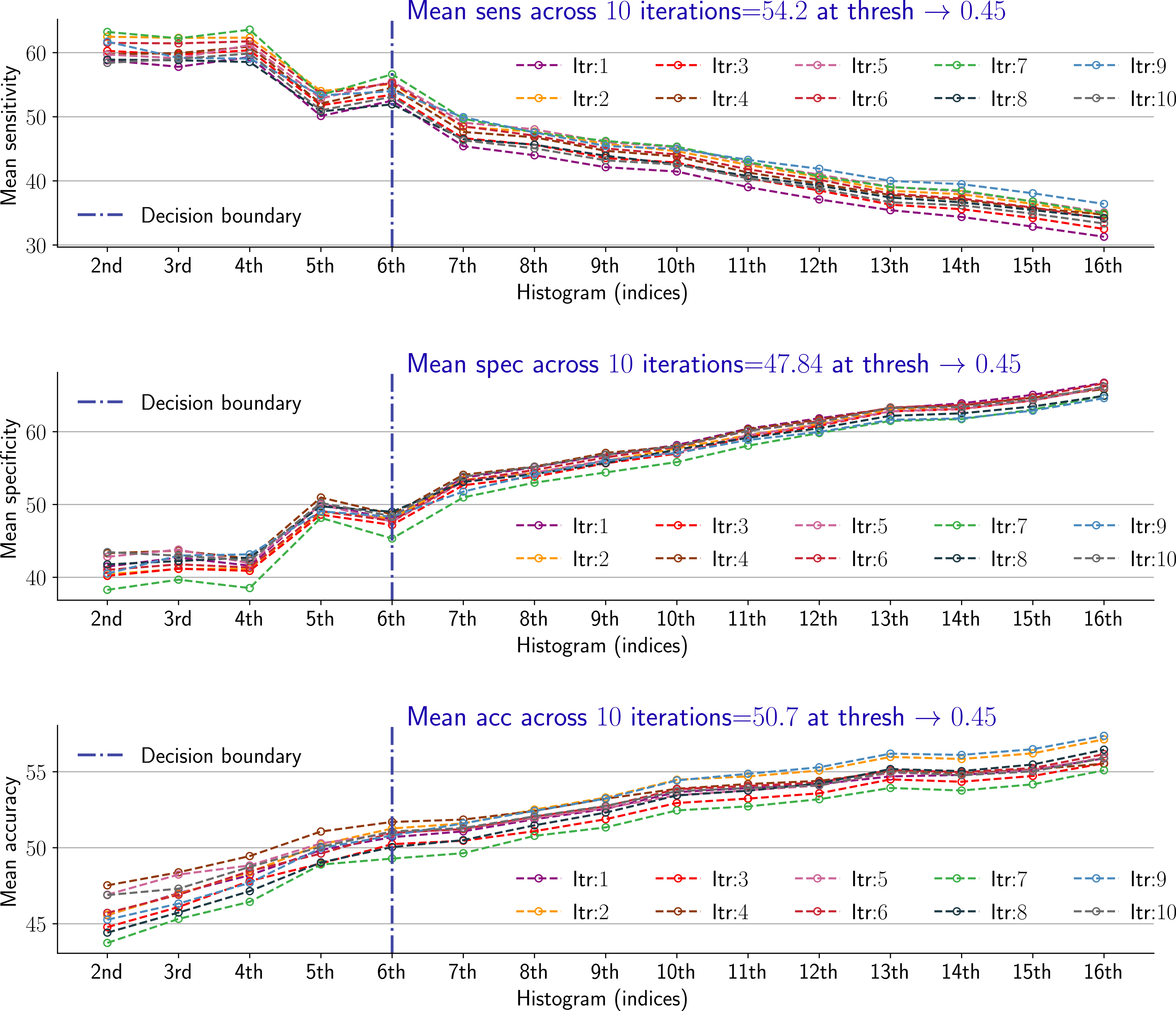

Supplement: Multimedia Appendix 2 [file jmir_v22i9e18297_app2.zip › high_quality_source_png_images_0002/Figure_0007.png]

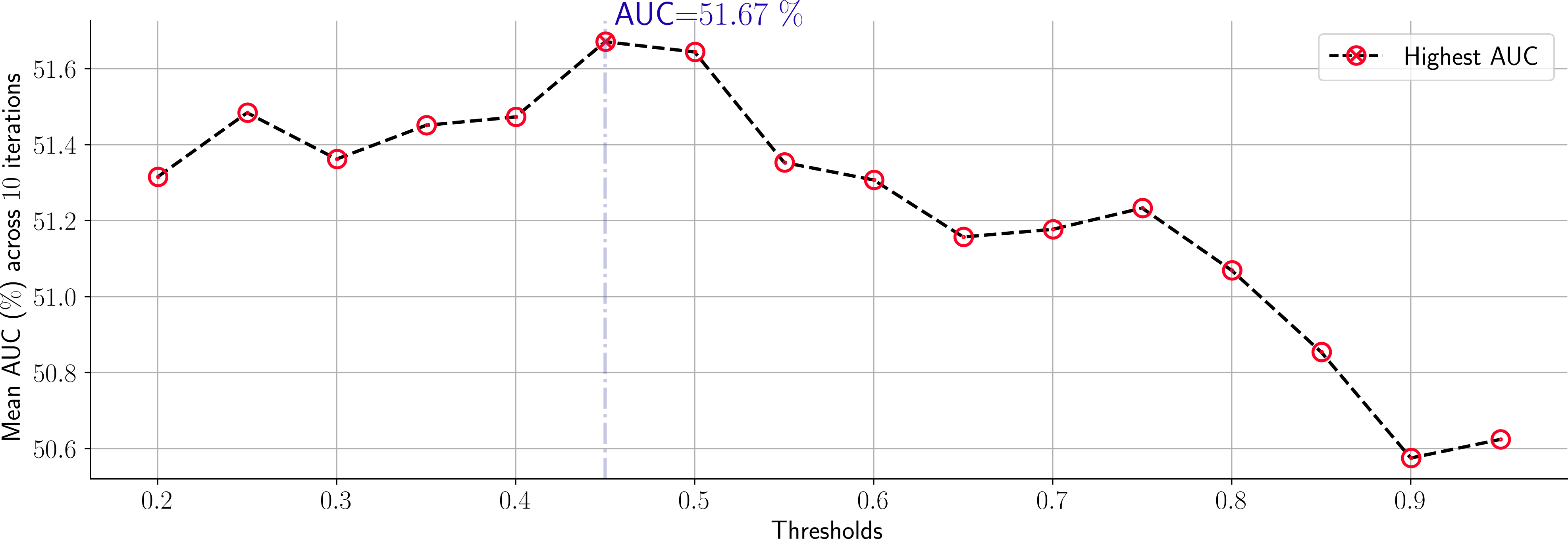

Supplement: Multimedia Appendix 2 [file jmir_v22i9e18297_app2.zip › high_quality_source_png_images_0002/Figure_0008.png]

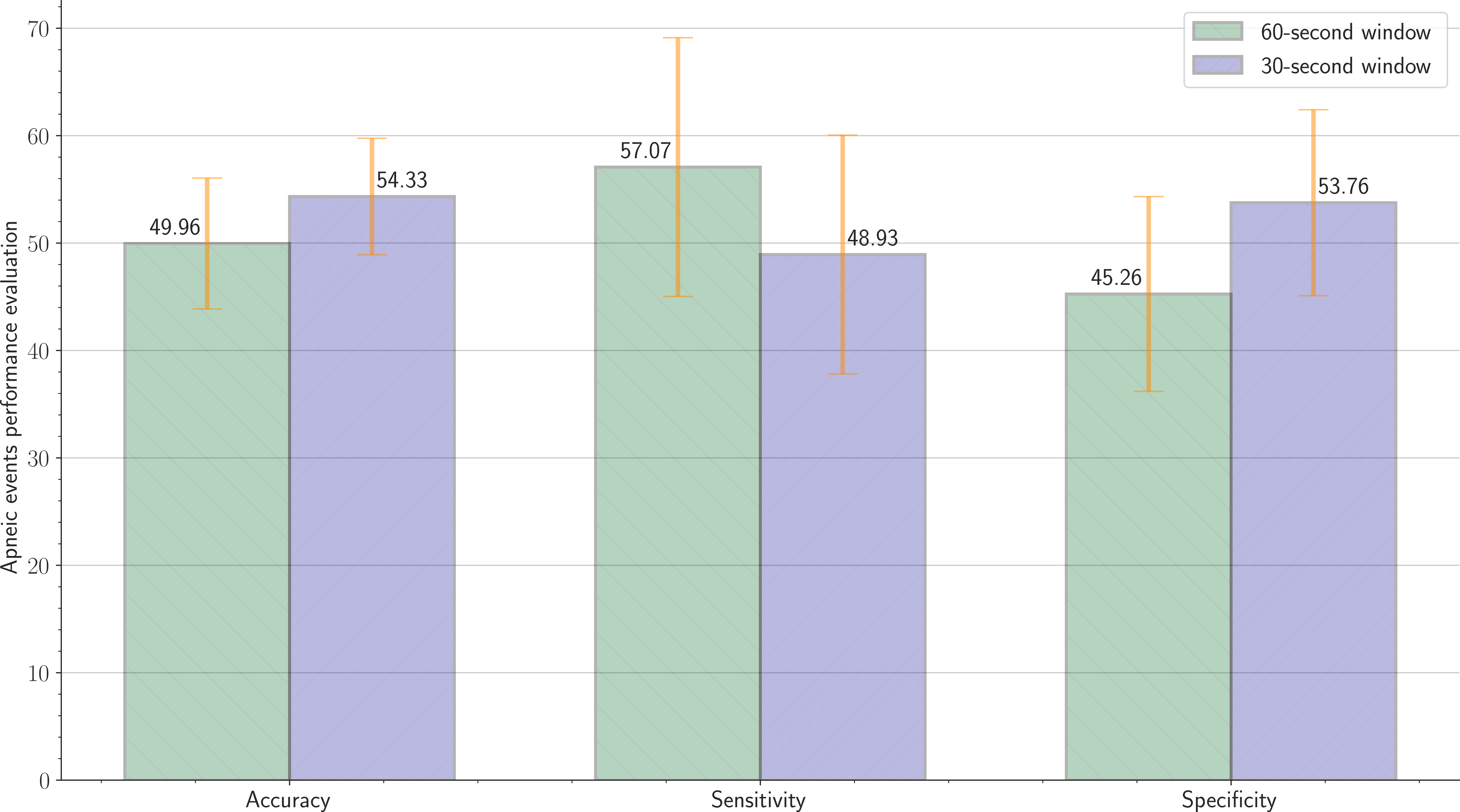

Supplement: Multimedia Appendix 2 [file jmir_v22i9e18297_app2.zip › high_quality_source_png_images_0002/Figure_0013.png]

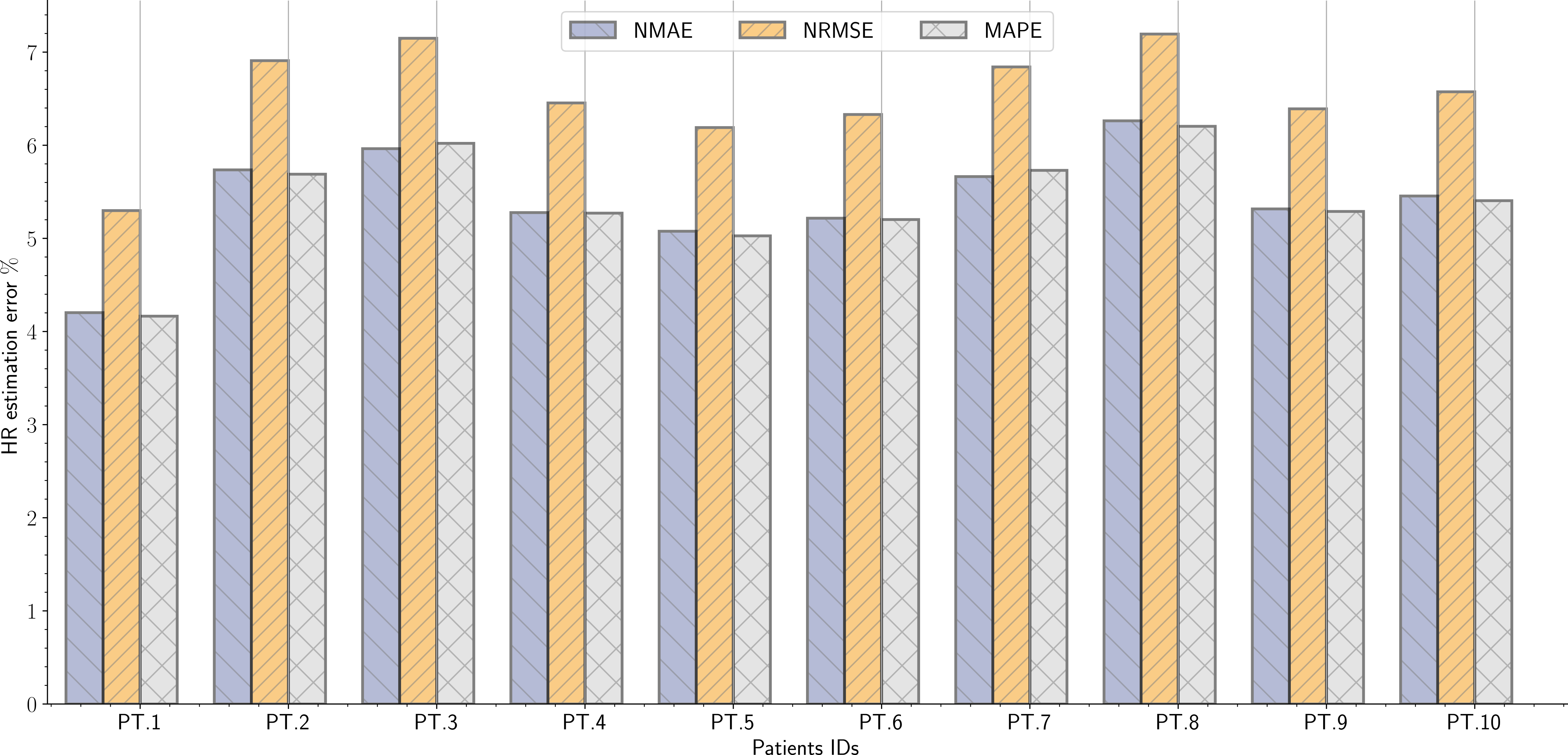

Supplement: Multimedia Appendix 2 [file jmir_v22i9e18297_app2.zip › high_quality_source_png_images_0002/Figure_0014.png]

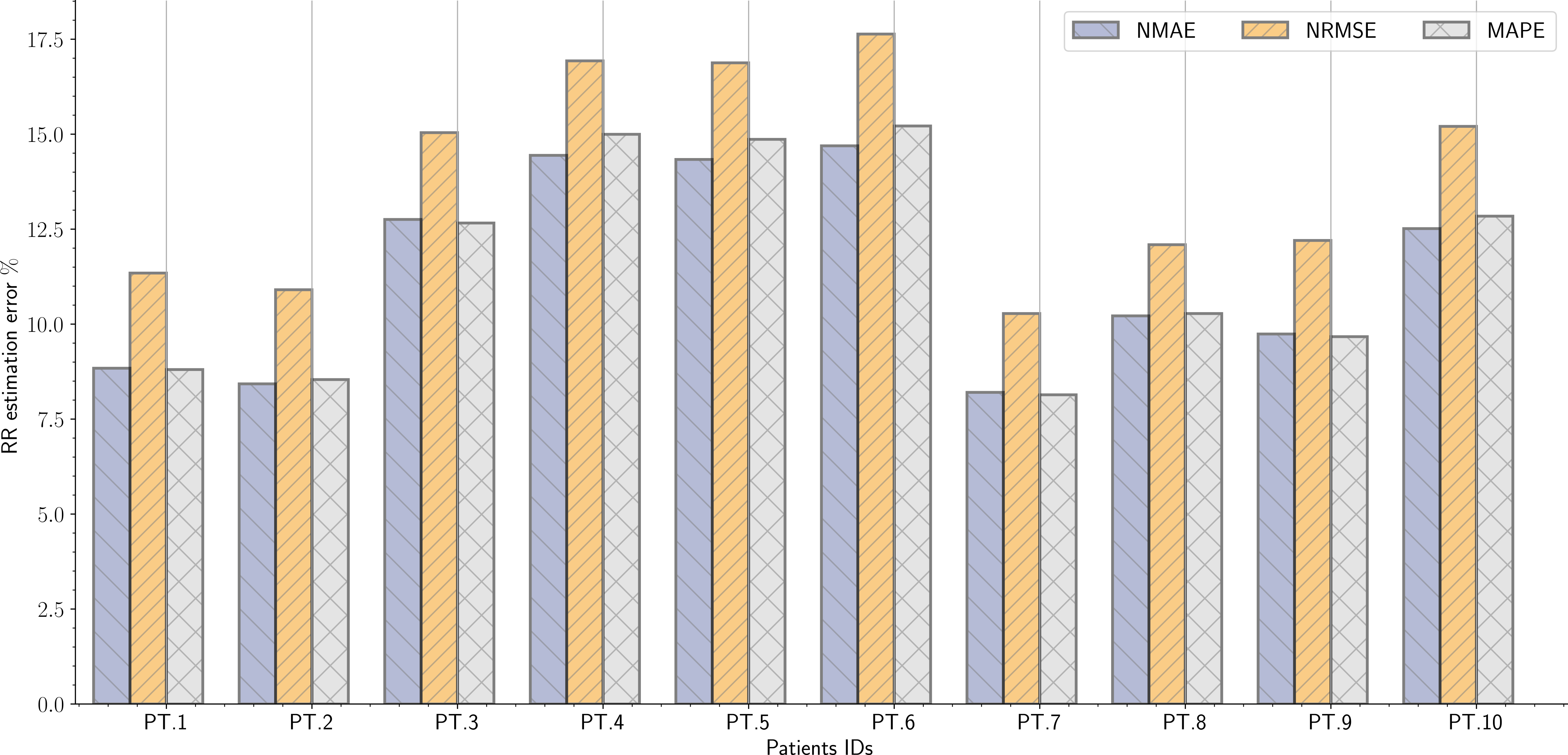

Supplement: Multimedia Appendix 2 [file jmir_v22i9e18297_app2.zip › high_quality_source_png_images_0002/Figure_0015.png]

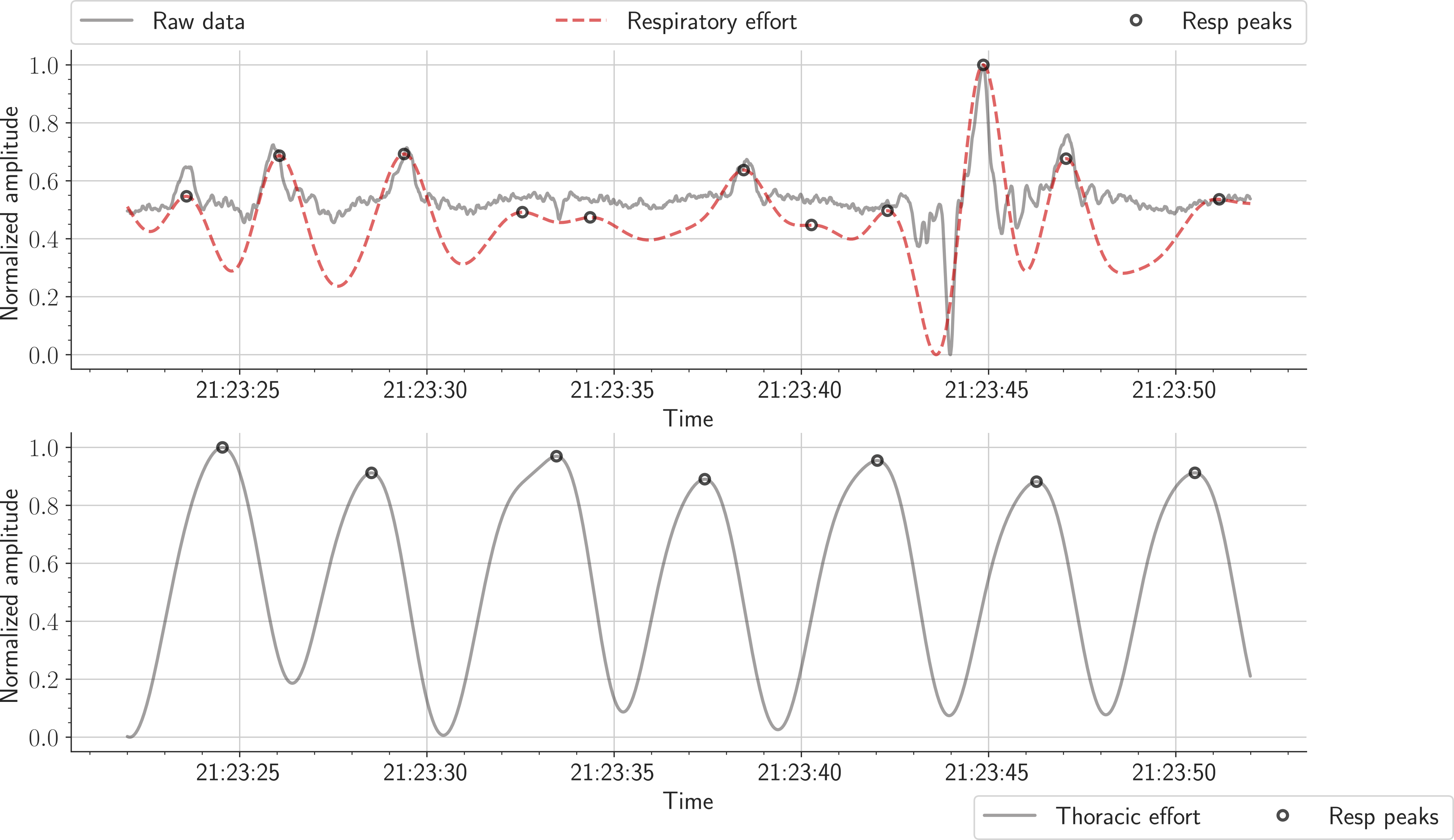

Supplement: Multimedia Appendix 2 [file jmir_v22i9e18297_app2.zip › high_quality_source_png_images_0002/Figure_0016.png]

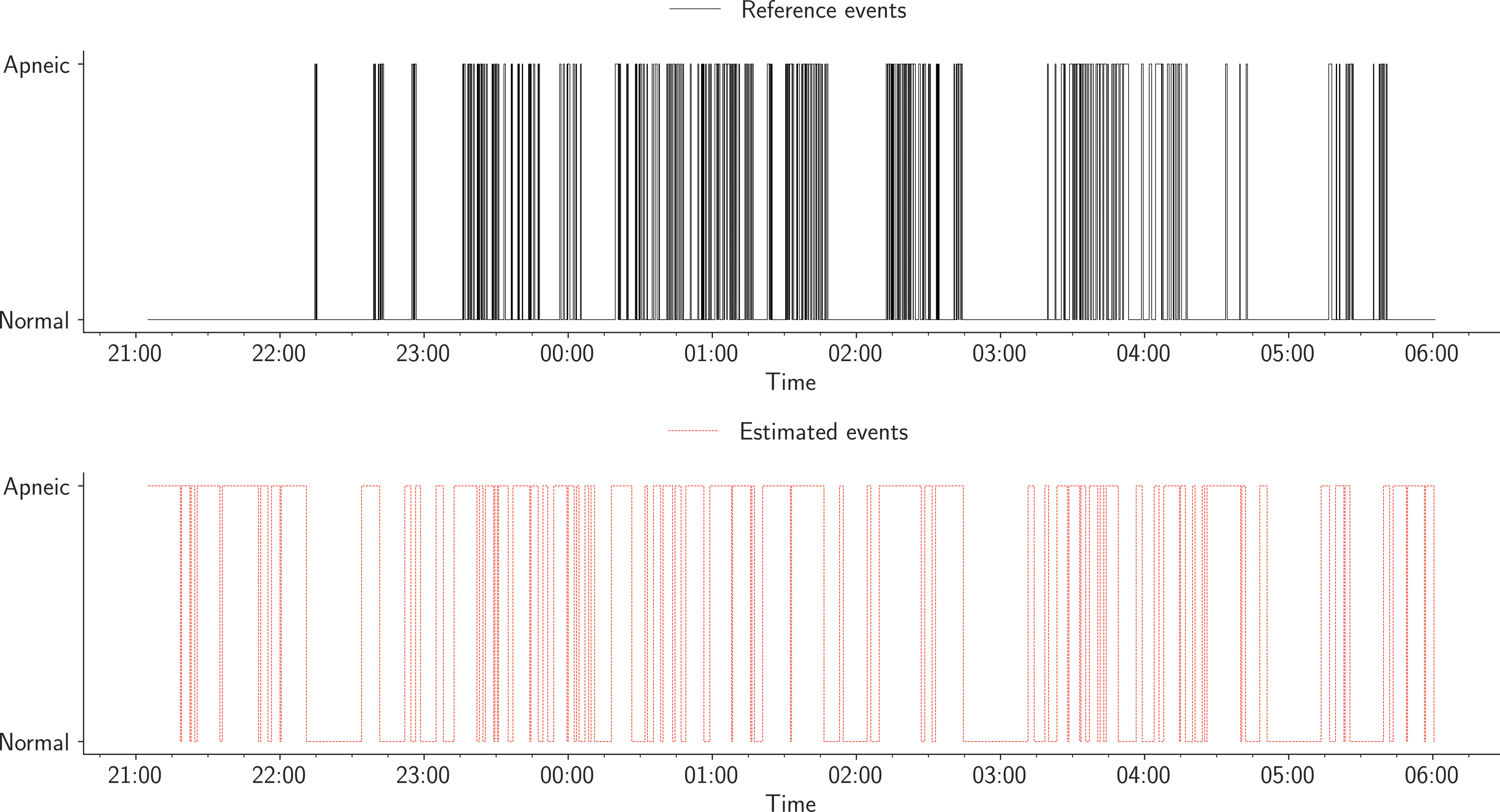

Supplement: Multimedia Appendix 2 [file jmir_v22i9e18297_app2.zip › high_quality_source_png_images_0002/Figure_0017.png]
